# The Role of Water in Carbon Dioxide Adsorption in Porphyrinic Metal‐Organic Frameworks

**DOI:** 10.1002/cctc.202300722

**Published:** 2023-08-08

**Authors:** Bettina Baumgartner, P. Tim Prins, Jaap N. Louwen, Matteo Monai, Bert M. Weckhuysen

**Affiliations:** ^1^ Debye Institute for Nanomaterials Science and Institute for Sustainable and Circular Chemistry, Department of Chemistry Utrecht University Universiteitsweg 99 3584 CG Utrecht The Netherlands

**Keywords:** Adsorption, artificial photosynthesis, CO_2_ capture, metal-organic framework, spectroscopy

## Abstract

Capturing and converting CO_2_ through artificial photosynthesis using photoactive, porous materials is a promising approach for addressing increasing CO_2_ concentrations. Porphyrinic Zr‐based metal‐organic frameworks (MOFs) are of particular interest as they incorporate a photosensitizer in the porous structure. Herein, the initial step of the artificial photosynthesis is studied: CO_2_ sorption and activation in the presence of water. A combined vibrational and visible spectroscopic approach was used to monitor the adsorption of CO_2_ into PCN‐222 and PCN‐223 MOFs, and the photophysical changes of the porphyrinic linker as a function of water concentration. A shift in CO_2_ sorption site and bending of the porphyrin macrocycle in response to humidity was observed, and CO_2_/H_2_O competition experiments revealed that the exchange of CO_2_ with H_2_O is pore‐size dependent. Therefore, humidity and pore‐size can be used to tune CO_2_ sorption, CO_2_ capacity, and light harvesting in porphyrinic MOFs, which are key factors for CO_2_ photoreduction.

## Graphical abstract

**MOFs combined with photochemistry** for CO_2_ photoreduction have great potential for addressing the challenge of reducing CO_2_ emissions. Herein, a combined visible‐infrared spectroscopic approach is reported to study the initial step of CO_2_ photoreduction: adsorption of CO_2_ in the presence of water into porphyrinic Zr‐MOFs. The findings will serve as guideline to tune adsorption site and photophysical properties of the photosensitizer.

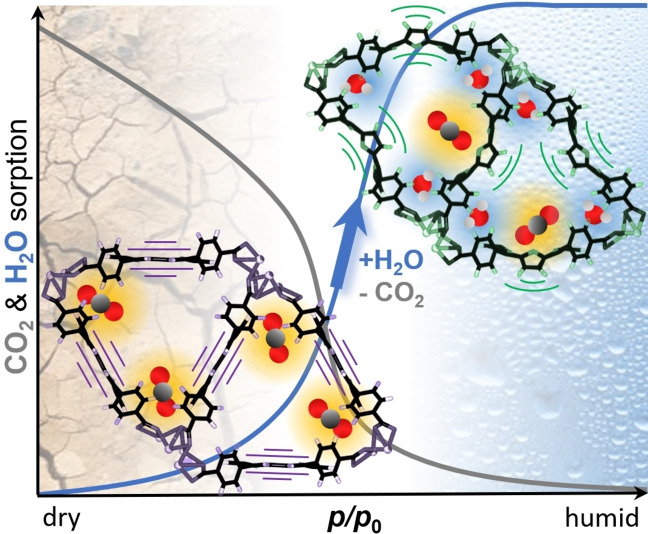

## Introduction

**Figure 1:**
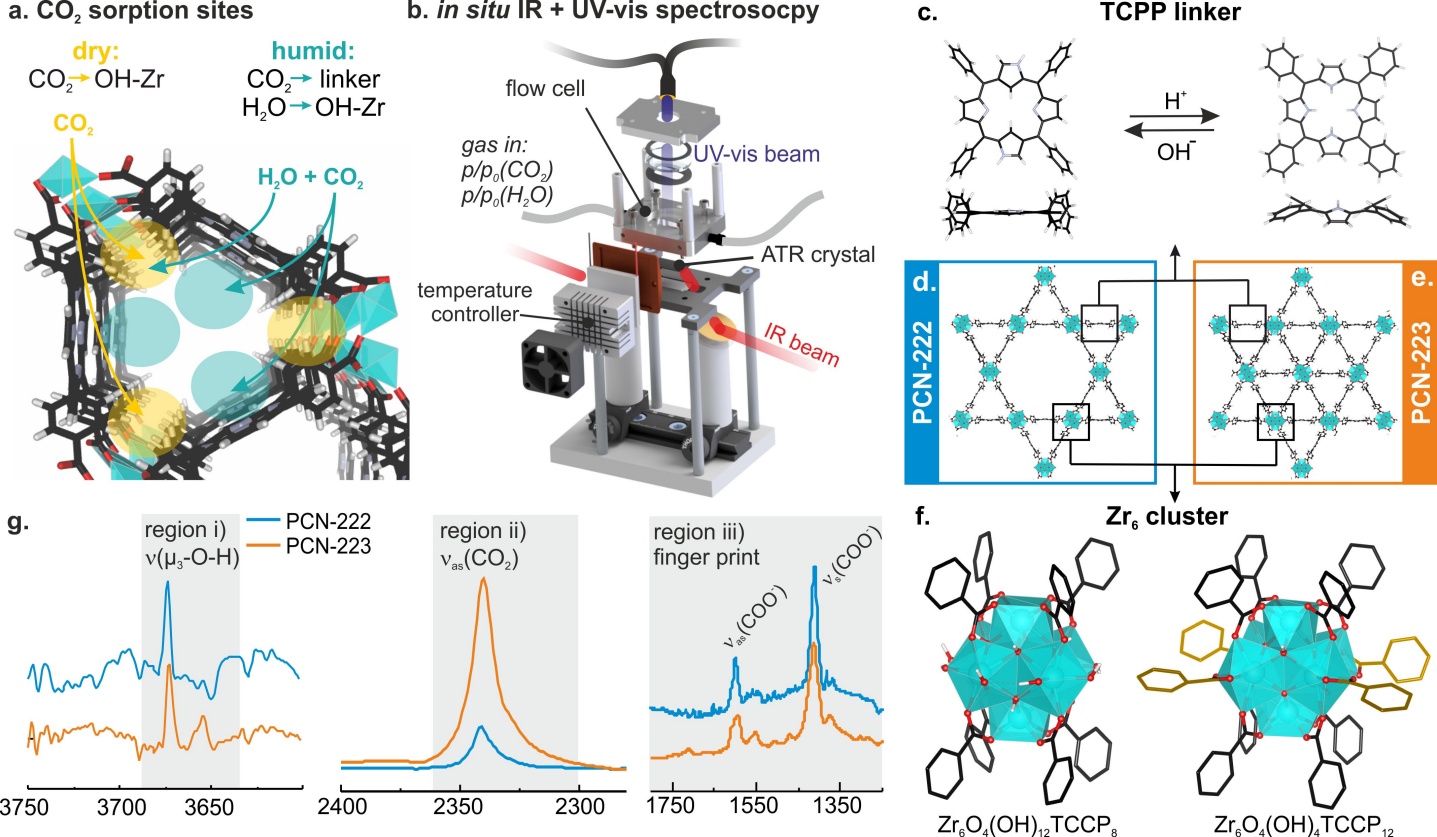
**a**. CO_2_ and H_2_O adsorption sites in the pores of metal‐organic frameworks (MOFs). **b**. Experimental setup combining *in situ* Fourier transform infrared‐attenuated total reflection (FT‐IR ATR) spectroscopy with ultraviolet‐visible (UV‐vis) spectroscopy in diffuse reflectance (DR) mode from the attenuated total reflection (ATR) crystal's surface. **c–e**. Crystal structure of PCN‐222 (d), PCN‐223 (e), and neutral and protonated structure of the porphyrin linker (c). **f**. Ball‐stick representation of the structure of the Zr‐clusters surrounded by 8 and 12 tetrakis(4‐carboxyphenyl)porphyrin (TCPP) linkers, respectively (only phenyl‐rings of TCPP are shown for simplicity). Linkers coordinated via the carboxylate to one Zr atom for PCN‐223 are highlighted in yellow. **g**. Regions of interest studied in the FT‐IR ATR spectra during CO_2_ adsorption into PCN‐MOFs and linker, including O−H stretching vibrations (region i)), asymmetric ν(C=O) stretching vibration of adsorbed CO_2_ (region ii)), and fingerprint region of the PCN‐MOF vibrations (region iii)) All spectra were subtracted with the blank Si ATR crystal background.

Metal‐organic frameworks (MOFs) are porous materials consisting of metal nodes and organic linkers. Due to their large surface areas and tunable porosity, MOFs have been extensively studied for gas separation and gas capture applications.[^1^, ^2^] In particular, the capture of the major greenhouse‐gas CO_2_ has gained significant attention and a vast number of MOFs have been investigated in terms of their CO_2_ sorption capabilities.[^3^, ^4^] In recent years, porphyrin‐based Zr‐MOFs have been added to the Zr‐based MOF family as efficient material for carbon capture.[^5^, ^6^, ^7^] Incorporating photoactive porphyrin‐dyes as linker into the framework is particularly attractive as it extends the MOF's scope of application to the photochemical conversion of the captured CO_2_ into e. g. formate, methane or methanol.[^8^, ^9^, ^10^, ^11^, ^12^, ^13^] Since water is often used as a source of protons,[^14^, ^15^] investigating competitive adsorption of CO_2_ and H_2_O on active sites is crucial to understand the CO_2_ activation and find the optimal reaction conditions to increase reaction rates in artificial photosynthesis. So far, only the externally applied CO_2_ and H_2_O concentrations have been regulated in closed photoreactors at the start of the photoreduction.[^14^, ^15^] Thereby, no insights into the actual CO_2_ and H_2_O concentrations in the MOF pores and how both adsorbates affect each other could be obtained. In addition, the optoelectronic properties of the porphyrin linker are highly sensitive to changes in solvent and acidity and have yet to be studied as function of adsorbed CO_2_ and H_2_O concentration. Interactions with water or intermediates of the photoreaction might cause the protonation of the imine‐N in the porphyrin macrocycle. This results in the disruption of the π‐electron conjugated system and the loss of planarity (Figure 1c),^[16]^ which in turn triggers a change in color from violet to green. This is of particular interest for photoreactions, as it affects the spectral overlap of the photoactive material with the excitation light source. In conjunction with the bending of the porphyrin macrocycle, the N−H groups become more accessible and their basicity increases, which has been shown to enhance catalytic activity.^[17]^ However, the influence of the protonation and structural changes of the porphyrinic linker on the photoreduction of CO_2_ are yet to be determined.

We suspect the interplay between CO_2_ and H_2_O sorption sites and the photophysical state of the porphyrin linker to be a key aspect in the photoreduction. Yet, their simultaneous observation was hampered by the lack of experimental equipment. Standard gas sorption experiments are limited to one adsorbate and co‐adsorption of binary systems, i. e., CO_2_ and H_2_O, can only be studied indirectly. Furthermore, no insights into photo‐physics of the MOF or linker can be obtained.

To overcome these limitations, we combined CO_2_ sorption with *in situ* Fourier transform‐infrared (FT‐IR) spectroscopy in attenuated total reflection (ATR) configuration and ultraviolet‐visible diffuse reflection spectroscopy (UV‐vis DRS) to directly investigate the co‐adsorption process of CO_2_ and H_2_O and its influence on the optoelectronic properties on photoactive MOFs (Figure 1b). We investigated the CO_2_ adsorption into two porphyrinic Zr‐MOFs, namely PCN‐222 and PCN‐223 (Figure 1c–f), consisting of Zr_6_O_4_(OH)_4_ clusters and tetrakis(4‐carboxyphenyl)porphyrin (TCPP) linkers in the absence and presence of water. These PCN‐MOFs were chosen as they consist of identical Zr‐clusters and porphyrin linkers, but differ in their pore size (micro‐ and mesoporous PCN‐222 and fully microporous PCN‐223, Figure 1d,e), which drastically influences the sorption characteristics and accessibility of the Zr‐cluster. In combination with density functional theory (DFT) simulations, we determined two CO_2_ sorption sites close to the Zr‐cluster and the porphyrin macrocycle, which depend on the presence of water (Figure 1a). *In situ* UV‐vis DRS revealed that the shift in sorption site is accompanied with a bending of the initially flat porphyrin macrocycle. Lastly, we have studied the competition between CO_2_ and H_2_O and found different exchange behaviors depending on the pore size and if the PCN‐MOF has been brought in contact with high humidity. These insights allow to match the CO_2_ sorption site with photoactive centers (either porphyrin or Zr‐cluster) as well as tuning the photo‐physics of the photosensitizer (porphyrin) during CO_2_ photoreduction in porphyrinic MOF materials.

## Results and Discussion

### Synthesis and Characterization of Metal‐Organic Frameworks

PCN‐222 and PCN‐223 based on tetratopic tetrakis(4‐carboxyphenyl)porphyrin (TCPP) and Zr‐clusters were synthesized solvothermally using different mediators.^[18]^ Both PCN‐MOFs consist of Zr_6_ clusters surrounded by 8 and 12 TCPP linkers, respectively, and differ in cluster connectivity (see Figure 1c–f). While in PCN‐222 all linkers are connected via a bridging of the carboxylate to two Zr atoms, in the structure of PCN‐223 additional four linkers are added to Zr−OH groups that are unoccupied in PCN‐222 (see Figure 1f, chelating carboxylates to only one Zr at highlighted in yellow). X‐ray diffraction (XRD) confirmed the formation of PCN‐222 and PCN‐223 phase‐pure crystal structures (ESI Figure S2). N_2_ sorption yielded Brunauer Emmett Teller (BET) surface areas between 2100–2340 m^2^ g^−1^. Type IV and type II isotherms were observed for PCN‐222 and PCN‐223 respectively, confirming the presence of both mesopores and micropores in PCN‐222 and only micropores in PCN‐223 (ESI Figure S3). The presence of the asymmetric and symmetric ν(COO^−^) band at 1606 cm^−1^ and 1420 cm^−1^ in the FT‐IR ATR spectra confirmed the bridging of the carboxylate of TCPP to two Zr atoms of the Zr‐cluster (Figure 1g, region iii), Figure S4).[^19^, ^20^] An additional band at 1551 cm^−1^ is visible in the region of the asymmetric carboxylate vibration in the spectrum of PCN‐223. We attributed this vibration to the different linker connectivity and the four TCPP linkers chelating to only one Zr atom in the Zr‐cluster. The FT‐IR ATR spectra of both PCN‐MOFs showed a band at 3673 cm^−1^ (Figure 1g) that corresponds to the μ_3_‐hydroxyl groups on the Zr‐cluster.[^20^, ^21^]

### CO_2_ Sorption onto Metal‐Organic Frameworks and Porphyrin Linker

**Figure 2:**
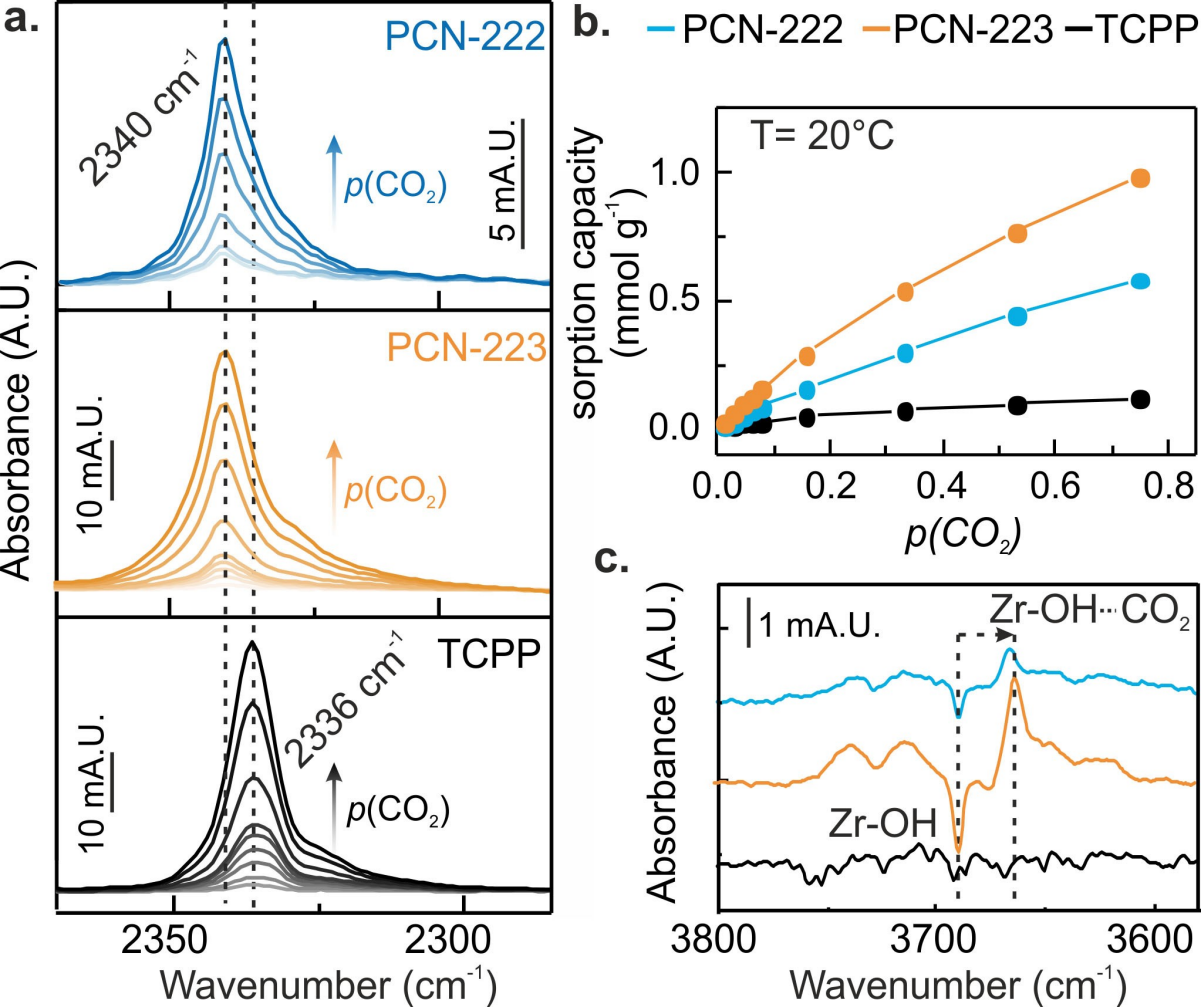
**a**. Fourier transform‐infrared (FT‐IR) attenuated total reflectance (ATR) spectra during CO_2_ adsorption for *p*(CO_2_)=0.02 bar to 0.75 bar into PCN‐222, PCN‐223 and TCPP, focusing on the ν(C=O) band region. Spectra were obtained with volume fractions of 2 %, 5 % and 13 % of PCN‐222, PCN‐223 and TCPP. Gas‐phase CO_2_ bands were subtracted to evidence adsorbed CO_2_ signals. **b**. CO_2_ sorption capacity for PCN‐222, PCN‐223 and TCPP derived from the ν_as_(C=O) band areas as function of applied CO_2_ concentration. **c**. FT‐IR ATR spectra of the Zr−OH bands region at *p*(CO_2_)=0.75 bar, showing a shift in the Zr−OH band of 30 cm^−1^, while no changes are visible for TCPP (background recorded at *p*(CO_2_)=0 bar).

PCN‐222 and PCN‐223 films were drop casted from acetone suspensions on Si ATR crystals and placed into a gas flow cell fixed in an FT‐IR spectrometer. A sapphire window on top of the flow cell allowed for fiber‐coupled UV‐vis DRS from the Si ATR crystal (see Figure 1b). Gases and vapors were supplied by means of mass flow controllers and the adsorption and desorption of CO_2_ into PCN‐222, PCN‐223 and TCPP was recorded using FT‐IR ATR spectroscopy (see Experimental Section and ESI for detailed description of the setup). The resulting FT‐IR ATR spectra can be divided into three regions of interest (Figure 1g): i) ν(μ_3_‐O−H) vibration of Zr−OH clusters between 3700–3600 cm^−1^, ii) the asymmetric stretching vibration of adsorbed CO_2_ around 2340 cm^−1^, and iii) the fingerprint region of the PCN‐MOF between 1800–1200 cm^−1^. Figure 2a shows the FT‐IR ATR spectra acquired during CO_2_ adsorption for PCN‐222, PCN‐223 and the free linker TCPP in region (ii) after subtraction of gas‐phase CO_2_ signals (Figure S5).

A strong ν_as_(C=O) band at 2340 cm^−1^ was observed for PCN‐222 and PCN‐223, which was red‐shifted with respect to the gas phase CO_2_ IR band at 2345 cm^−1^ due to the interaction of physiosorbed CO_2_ with the PCN‐MOF framework. In contrast, the ν_as_(C=O) band of CO_2_ adsorbed onto TCPP was found at 2336 cm^−1^. Both ν_as_(C=O) positions have been observed in UiO‐66:[^22^, ^23^] The band at 2340 cm^−1^ has been assigned to CO_2_ located close to the μ_3_‐OH groups of the Zr‐cluster, while the band at 2336 cm^−1^ appeared after full dihydroxylation of UiO‐66, i. e., complete removal of Zr−OH groups. The latter band has been assigned to physiosorbed CO_2_ stabilized through purely dispersive interaction with the π‐system of the terephthalic acid linkers.^[6]^ In addition, we concluded that only weak interactions between PCN‐MOF and CO_2_ take place, as evidenced by the absence of coordinated species typically observed in MOFs with open metal sites at wavenumbers higher than 2340 cm^−1^.^[24]^ We calculated the CO_2_ adsorption isotherms at 20 °C using the ν_as_(C=O) band areas as function of the applied CO_2_ concentrations (Figure 2b). The observed CO_2_ adsorption capacities of 0.6 mmol g^−1^ and 1.0 mmol g^−1^ at *p*(CO_2_)=0.75 bar for PCN‐222 and PCN‐223, respectively, were in line with values reported in literature (Figure 2b, see ESI for further explanation) and the fact that PCN‐222 hosts half the microporous CO_2_ sorption sites compared to PCN‐223.[^5^, ^25^, ^26^, ^27^] For the non‐porous, free linker, we retrieved a sorption capacity of 0.1 mmol g^−1^ at *p*(CO_2_)=0.75 bar, indicating that porosity and/or interaction with Zr clusters is necessary to adsorb CO_2_ in the studied PCN‐MOFs. Accordingly, a 30 cm^−1^ red‐shift of the Zr−OH band at 3673 cm^−1^ (Figure 2c) was observed during exposure to CO_2_ in both PCN‐MOFs, which we attribute to CO_2_⋅⋅⋅HO−Zr interactions.^[28]^ Note that this band shift was not visible in the TCPP FT‐IR ATR spectrum as no free Zr−OH‐groups are present in the free linker. Lastly, FT‐IR ATR spectra obtained during CO_2_ adsorption showed minor changes in the MOF fingerprint region, which indicated negligible interactions of the adsorbed CO_2_ molecules with the PCN‐MOF framework (Figure S6). This was in line with the UV‐vis spectra obtained upon CO_2_ adsorption that showed no spectral changes (Figure S7).

**Figure 3:**
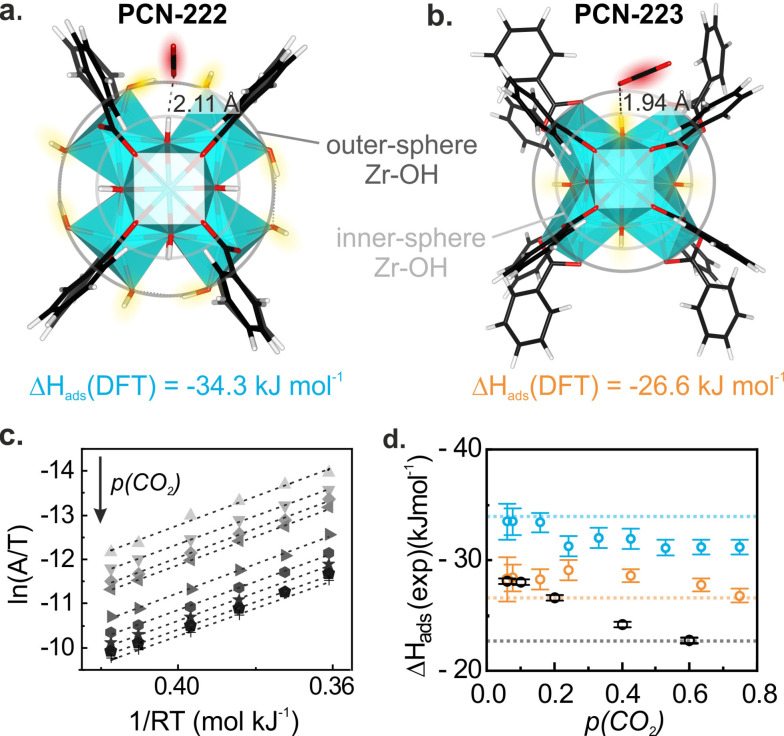
CO_2_ sorption sites at the PCN‐222 and PCN‐223 Zr‐cluster calculated by Density Functional Theory (DFT): **a**. Zr‐cluster of PCN‐222: CO_2_ interacts with the unoccupied, outer‐sphere Zr−OH groups (highlighted in yellow) and is stabilized in the pocket of the phenyl‐rings of the linkers (here simplified as benzoic acid moieties). **b**. Zr‐cluster of PCN‐223: CO_2_ interacts with the Zr−OH groups closer to the center of the cluster (inner‐sphere, highlighted in yellow) and is also stabilized by the aromatic system. **c**. Linearized plot of CO_2_ adsorbed into PCN‐222 for *p*(CO_2_)=0.04 bar to 0.75 bar at 15–60 °C for the van't Hoff equation derived from absorbance (*A*), ideal gas constant (R) and temperature (*T*), where the slope corresponds to *ΔH_ads_*. **d**. Adsorption enthalpy *ΔH_ads_* as function of applied CO_2_ concentration. Dashed lines correspond to the simulated values.

The variations in the ν_as_(C=O) band position of adsorbed CO_2_ in the free linker and PCN‐MOFs, shown in Figure 2a, indicated that CO_2_ adsorbed on different adsorption sites. We performed DFT simulations to gain further insights into the nature and strength of the CO_2_ binding sites in the PCN‐MOFs and the free linker (see Experimental Section for computational details). The calculated sorption site of CO_2_ on the Zr‐cluster representative of PCN‐222 and PCN‐223 are depicted in Figure 3a and b, respectively, where TCCP linkers were represented by benzoic acid moieties with the C‐atoms in para position to the carboxylate groups fixed at the position in the solid to facilitate the calculations (Figure S8 for more detailed view). Since the phenyl‐rings of the TCCP linker are rotated out of the plane of the porphyrin molecule in the solid, there is little or no electron delocalization between the porphyrin and the phenyl moieties. Hence, not including the porphyrin macrocycle was not expected to have a significant influence on the computational results. According to the models, CO_2_ molecules interact with free OH‐groups of the Zr‐clusters in both PCN‐MOFs, and are further stabilized in the pocket formed by the phenyl‐rings of the linkers. Note that the stabilization is due to a combination of van der Waals interactions as well as hydrogen bonding between CO_2_ and Zr−OH groups (Figure S8). Such a contribution of dispersion stabilization has been reported previously for similar Zr‐based MOFs and similar distances between the oxygen atom of CO_2_ and the HO−Zr group of 2.36 Å were found.[^3^, ^22^, ^23^] The different Zr‐cluster connectivity of PCN‐222 and PCN‐223 led to slightly different sorption sites: While CO_2_ interacts with the free outer‐sphere Zr−OH groups present in the cluster of PCN‐222, these Zr−OH groups are occupied by the linkers in PCN‐223. Therefore, CO_2_ interacts with the inner‐sphere Zr−OH groups closer to the center of the cluster. In addition, the distance between the carbon atom of CO_2_ and phenyl‐groups is larger in PCN‐223 compared to PCN‐222 (Figure S8). Figure S9 illustrates the CO_2_ sorption site on the free base TCPP linker. The CO_2_ molecule is stabilized close to the center of the porphyrin macrocycle, however, the distance between CO_2_ and N−H groups in the porphyrin core exceeds the typical length for H‐bonding. Consequently, no significant changes were experimentally observed in the fingerprint region of FT‐IR ATR spectra and the UV‐vis spectra upon CO_2_ sorption on the TCPP linker (Figure S6, S7).

To validate our models, we calculated the ν_as_(C=O) band positions of CO_2_ adsorbed on PCN‐MOFs (2338 cm^−1^) and the free TCPP linker (2335 cm^−1^). These values correlated well with the experimentally observed band positions, 2340 and 2336 cm^−1^ for the PCN‐MOFs and TCCP respectively (Figure 2a), and are in accordance with previously reported values on a similar MOF (i. e. UiO‐66).^[23]^ Moreover, to further verify the CO_2_ sorption site, we compared the CO_2_ adsorption enthalpy determined from temperature‐dependent CO_2_ sorption experiments and via computational methods (Figure 3c,d). Experimentally, we used the absorbance (*A*) of ν_as_(C=O) at temperatures between 15 to 60 °C to calculate the CO_2_ adsorption enthalpy *ΔH_ads_* based on the van ‘t Hoff equation.^[29]^ This method is based on the assumption that *A* is proportional to *ΔH*_*ads*,_ which is the case for low CO_2_ coverages. To justify this assumption, we determined the average number of CO_2_ molecules per Zr‐cluster using the sorption capacity of the PCN‐MOFs (Figure 2b) and divided this value by the theoretical Zr‐cluster density in the crystal structure of the PCN‐MOFs. Thereby, we found that 0.8 and 0.7 CO_2_ molecules per Zr‐cluster were present at the highest CO_2_ concentration (*p*(CO_2_)=0.75 bar) in PCN‐222 and PCN‐223, respectively.

The *ΔH_ads_* (CO_2_) was derived from the slope of the linearized plot at different CO_2_ pressures (Figure 3c, Figure S11). CO_2_ adsorption enthalpies of −31, −28 and −23 kJ mol^−1^, for PCN‐222, PCN‐223 and TCPP at *p*(CO_2_)=0.75 bar were thereby obtained (Figure 3d). Furthermore, similar trends as function of *p*(CO_2_) and *ΔH_ads_* values have been reported for UiO‐66.[22,23] Due to the low CO_2_ loading in TCPP, a plateau of *ΔH_ads_* was not reached in the given *p*(CO_2_) range but *ΔH_ads_* converged to the simulated value for the highest CO_2_ pressure. Due to the convergence observed for TCPP and the fact that only the highest *p*(CO_2_) yielded a number of CO_2_ molecules per Zr‐cluster close to one, which was used for the computational considerations (Figure 3a,b), we chose to compare the experimentally and computationally obtained *ΔH_ads_* values at the highest CO_2_ pressure (*p*(CO_2_)=0.75 bar). At this partial pressure, the experimentally obtained *ΔH_ads_* agreed with the DFT simulated adsorption energy of −34.3, −26.6, and −22.9 kJ mol^−1^ for PCN‐222 cluster, PCN‐223 cluster and TCPP, respectively. Note that, even though a greater redshift of the dispersion‐stabilized band at 2336 cm^−1^ compared to the interaction with the Zr‐cluster would indicate a stronger interaction between CO_2_ and PCN‐MOF, and thus a lower adsorption enthalpy, at the first glance, other effects such as change in bond length or angle of the CO_2_ molecule play a role in the band position as well.^[30]^ The excellent agreement of both the adsorption enthalpy and band position, and the found shifts in Zr−OH band (Figure 2c) confirm the sorption site of CO_2_ into PCN‐222 and PCN‐223 close to the Zr‐cluster as shown in Figure 3a‐b.

### Water Adsorption into PCN‐222 and PCN‐223

**Figure 4:**
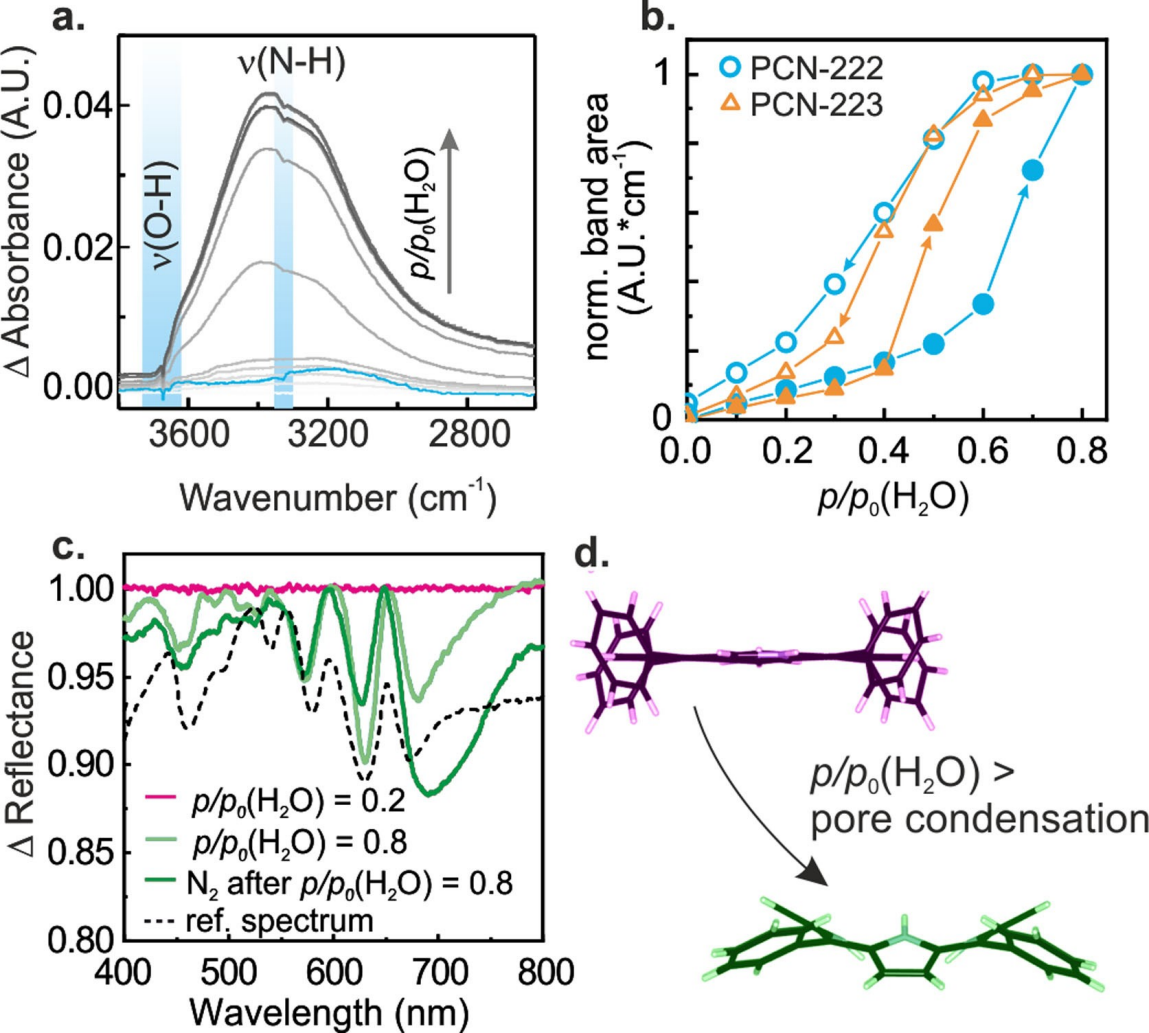
Water sorption into PCN‐222 and PCN‐223. **a**. Fourier transform‐infrared (FT‐IR) attenuated total reflection (ATR) spectra of the ν(OH) region recorded with PCN‐223 for *p/p*_0_(H_2_O)=0–0.8. The blue spectrum was recorded following water adsorption at *p/p*_0_(H_2_O)=0.8, after 10 min N_2_ flushing, and shows the irreversible change as ν(O−H) and ν(N−H) negative bands of the Zr−OH groups and porphyrin macrocycle, respectively (highlighted in blue). A band at 3200 cm^−1^ was assigned to minor amounts of residual adsorbed water. **b**. Water isotherms were derived from the integral of the ν(O−H) stretching region between 2800–3600 cm^−1^. Full symbols correspond to the adsorption branch of the isotherm, while empty symbols correspond to the desorption branch. **c**. Ultraviolet‐visible (UV‐vis) diffuse reflectance spectroscopy (DRS) data during water sorption into PCN‐223 and subsequent N_2_ flushing. Irreversible changes in the UV‐vis DRS data were only visible if *p/p*_0_(H_2_O)>pore condensation was applied. For PCN‐222 spectra we refer to Figure S16. **d**. Schematic of structural changes of the porphyrin linker upon protonation. Less bending is expected for wetting.

Water vapor was applied at different partial pressures *p/p*_0_(H_2_O)=0–0.8, and FT‐IR ATR and UV‐vis spectra were recorded for each *p/p*_0_(H_2_O) to determine the interaction of the PCN‐MOF structure with adsorbing water. The FT‐IR ATR spectra of both PCN‐MOFs showed a gradual increase of bands between 3200–3400 cm^−1^ and at 1640 cm^−1^, that correspond to adsorbed water (Figure 4a, Figure S12‐13). Although the adsorbed water bands were mostly removed upon flushing with dry N_2_ (Figure 4b, blue spectrum), an irreversible general broadening of the IR bands corresponding to the PCN‐MOFs was observed (Figure S12). The absence of an increase in bands associated with free COOH‐moieties of the linker (typically found around 1700 cm^−1^) indicates that no unattached linkers, i. e. defects, were formed during wetting. In addition, a sharp negative band at 3673 cm^−1^ appeared that was not recovered upon N_2_ flushing (Figure S13). This band indicates the irreversible bonding of water to the Zr‐cluster, unaffected by N_2_ flushing at room temperature. This experimental finding was supported with DFT simulations that yielded a considerably higher adsorption enthalpy of water compared to CO_2_, of −74.7 and −52.6 kJ mol^−1^ for PCN‐222 and PCN‐223 (*vs*. −34.3 and −26.6 kJ mol^−1^ for CO_2_), respectively. In addition to ν(μ_3_‐O−H), a negative band at 3324 cm^−1^ appeared at *p/p*_0_(H_2_O) >0.6 and >0.4 for PCN‐222 and PCN‐223, respectively, which was also irreversible upon N_2_ flushing, and was associated with the ν(N−H) band in the porphyrin macrocycle. Distortions of the ν(N−H) band upon interaction with H_2_O and CO_2_ have been observed in MOFs with linkers with amine‐moieties.^[31]^ The appearance of the ν(μ_3_‐O−H) and ν(N−H) bands coincided with a steep increase in the water sorption isotherms, which were obtained from the integral of the entire ν(O−H) region of the FT‐IR ATR spectra and are given in Figure 4b. The water concentration shows a sharp rise at *p/p*_0_(H_2_O)=0.6 and 0.4 for PCN‐222 and PCN‐223, respectively. This indicates the the condensation of water in the pores, which depends on the pore size. Smaller pores lead to earlier pore condensation, resulting in a lower onste at *p/p*_0_(H_2_O) for the microporous PCN‐223 compared to the mesoporous PCN‐222.^[32]^ The shift of the ν(O−H) band maximum from 3200 cm^−1^ to 3400 cm^−1^ upon pore condensation further indicates the presence of non‐surface‐bound, liquid‐like water, which is mainly present in the case of fully filled pores.[^32^, ^33^] Non‐closing isotherms indicated that slight amounts of water remained in the pores after flushing with N_2_. Note that, when a *p/p*_0_(H_2_O) below the critical pressure for pore condensation is applied, the FT‐IR ATR spectral changes of the OH‐groups of the Zr‐cluster were reversible and no changes in the ν(N−H) band were observed (Figure S14). These findings suggest that irreversible binding of water and structural changes of the PCN‐MOF framework only took place upon entire pore filling by condensed water.

The observations in the FT‐IR ATR spectra correlated with the spectral features found using *in situ* UV‐vis spectroscopy: For *p/p*_0_(H_2_O)<pore condensation, small bands between 600–700 nm could be reversed upon N_2_ flushing (Figure S16). For *p/p*_0_(H_2_O)>pore condensation very strong differential signals between 450–750 nm were visible, which did not recover upon N_2_ flushing (Figure 4c for PCN‐223, Figure S16 for PCN‐222). A series of reference experiments indicated that the UV‐vis spectra are due to a change in the sample refractive index during pore condensation, and to the protonation of pyrrole moieties in the porphyrin, which causes a bending of the macrocycle previously observed for the TCPP linker and PCN‐222 upon acidification (see Figure S17 and discussion in the ESI).[^16^, ^34^, ^35^]

That a partial porphyrin protonation takes place upon condensation of water in the PCN‐MOFs pores is a very peculiar finding, as the bending of the porphyrin macrocycle is typically only reported for protonated TCPP species obtained by the addition of acid. XRD and N_2_ physisorption confirmed that the structural changes are limited to the linker, while the PCN‐MOF structures remained intact (see Figure 4d, Figure S18–19).

### CO_2_ Sorption on Pre‐Wetted Metal‐Organic Frameworks

**Figure 5:**
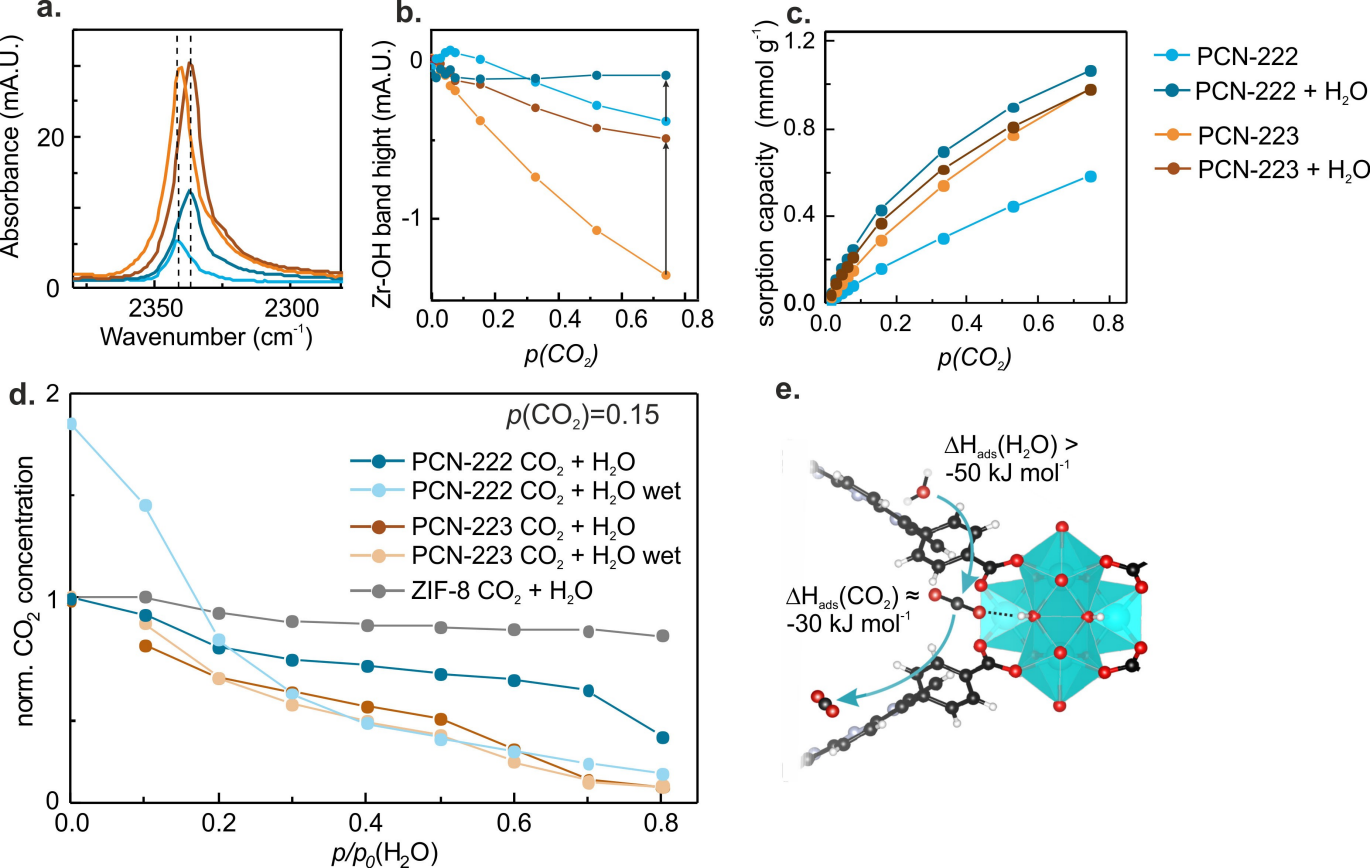
CO_2_ sorption on wetted PCN‐222 and PCN‐223. **a**. Fourier transform‐infrared (FT‐IR) attenuated total reflection (ATR) spectrum showing the ν_as_(C=O) band on wetted and pristine PCN‐222 and PCN‐223 during CO_2_ adsorption at *p*(CO_2_)=0.75 bar. **b**. ν(μ_3_‐OH) band of Zr−OH as function of CO_2_ concentration for pristine and wetted PCN‐222 and PCN‐223. **c**. CO_2_ sorption capacities for pristine and wetted metal‐organic frameworks (MOFs) derived from the band area of the ν(C=O) band. **d**. Adsorbed CO_2_ concentration into PCN‐222, PCN‐223 and ZIF‐8 as a function of water partial pressure. CO_2_ concentrations were normalized to CO_2_ adsorption values recorded at *p/p*_0_(H_2_O)=0. **e**. Ball‐stick representation of the competitive adsorption of water and CO_2_.

Having confirmed that water sorption causes irreversible structural changes in the linkers of PCN‐MOFs, we determined the influence of these changes on the CO_2_ sorption sites and the CO_2_ sorption capacity of PCN‐222 and PCN‐223. After applying *p/p*_0_(H_2_O)>pore condensation and flushing with N_2_, we collected CO_2_ isotherms at different temperatures, to derive the CO_2_ adsorption enthalpies analogous to the previous section. Thereby we found four significant changes compared to the CO_2_ sorption into pristine PCN‐MOFs: i) the ν_as_(C=O) band position shifted from 2340 cm^−1^ in the pristine case to 2336 cm^−1^ in the case of wetted PCN‐MOFs (Figure 5a); ii) the band intensity of the ν(μ_3_‐O−H) band at 3673 cm^−1^ decreased less as a function of CO_2_ pressure, compared to the CO_2_ sorption into the pristine PCN‐MOFs (Figure 5b); iii) the sorption capacity for PCN‐222 was enhanced 1.8‐fold and was now similar to PCN‐223, while the sorption capacity of PCN‐223 remained unchanged (Figure 5c); and iv) the adsorption enthalpies decreased from −31 to −22 kJ mol^−1^ for PCN‐222 and from −28 to −24 kJ mol^−1^ for PCN‐223 at *p*(CO_2_)=0.75 bar. A decrease in adsorption enthalpy alongside an increase in sorption capacity as found for PCN‐222 may seem counterintuitive. However, as we will explain in detail later, we attribute this behaviour to the presence of newly formed sorption sites. In contrast, we did not observe any changes for the wetted TCPP linker.

After wetting, the obtained CO_2_ adsorption enthalpies and ν_as_(C=O) band positions matched the values obtained for the free linker. This indicated that, after pore condensation with water, a shift in sorption site from the Zr‐cluster to the porphyrin macrocycle occurred (Figure 5e). This was further supported by the smaller change in Zr−OH band intensity upon CO_2_ adsorption compared to the pristine case, which stems from the interaction of the OH‐group with CO_2_ (Figure 5b). This suggested that these sites were occupied by water molecules after water adsorption, and were thus not accessible for CO_2_ anymore. No changes in UV‐vis spectra upon CO_2_ sorption into both wetted PCN‐MOFs were visible (Figure S20). As the color of the porphyrin linker is highly sensitive to changes in pH value, we expected a change in the UV‐vis spectra if carbonic acid would have formed from water and CO_2_. The absence of spectral changes as well as no corresponding FT‐IR bands in the fingerprint region of the FT‐IR ATR spectra suggested that no carbonates or carbonic acid formed and that water and CO_2_ remained unchanged in the pores.

We assign the increase in sorption capacity of PCN‐222 to newly generated CO_2_ sorption sites within the mesopores. This has been observed for other MOFs,^[36]^ including UiO‐66, where CO_2_ only adsorbs into the small tetrahedral cages (ca. 0.8 nm) for the pristine MOF and only after wetting, sorption sites in the larger octahedral cages (ca. 1.1 nm) can be occupied by CO_2_ and the sorption capacity increased by 20 %.[^37^, ^38^] Similarly, mesoporous MIL‐100 showed a 5‐fold increase in CO_2_ capacity after wetting with H_2_O and a decrease in adsorption enthalpy, which was ascribed to microporous pockets formed by pre‐adsorbed water in the mesopores of MIL‐100, which are attractive for CO_2_ sorption.^[36]^

In line with previous findings, we here propose that water opens up new sorption sites in the 3.6 nm mesopores of PCN‐222, most likely close to the porphyrin macrocycle, and therefore yields a higher sorption capacity and a lower adsorption enthalpy. In contrast, PCN‐223 has a higher sorption capacity already in the pristine state, which we attribute to the higher microporosity (only 1.1 nm sized‐pores) compared to PCN‐222. Therefore, we assume that no new pore spaces could be generated that would have allowed to accommodate more CO_2_ molecules. To further support the claim that the sorption site is influenced by humidity‐induced structural changes in the PCN‐MOF, we performed CO_2_ sorption in PCN‐222 treated with *p/p*_0_(H_2_O)=0.2 and 0.4, i. e. before water condensation. This did not cause any changes in CO_2_ sorption capacity or ν_as_(C=O) band position (Figure S14). Furthermore, CO_2_ sorption experiments performed on pre‐acidified PCN‐222 with 0.01 M HCl yielded identical sorption capacities and band positions as obtained for the fully wetted PCN‐MOF, which suggested that protonation of the PCN‐MOFs occurs after condensation from the gas phase (Figure S21).

### Competitive CO_2_/H_2_O Sorption

Lastly, we studied the competition of sorption sites between CO_2_ and H_2_O. To this end, increasing water vapor pressures were applied in the presence of a fixed CO_2_ pressures at *p*(CO_2_)=0.15 bar, which represents a typical flue gas concentration. The thereby obtained concentrations measured as the band areas of adsorbed CO_2_ (ν_as_(C=O)) and H_2_O (ν(O−H)) are given in Figure 5d and S21. Figure 5d shows the influence of increasing water vapor pressure on adsorbed CO_2_ for pristine (dark blue and brown traces) and wetted (light blue and beige traces) PCN‐MOFs. For the pristine PCN‐MOFs, a gradual decrease in CO_2_ concentration was found up to *p/p*_0_(H_2_O)=0.7 and 0.5 for PCN‐222 and PCN‐223, respectively, which correspond to the water pore condensation onsets for each PCN‐MOF (Figure 4b). After water condensation in the PCN‐MOF pores, the CO_2_ concentration decreased to 32 % and 8 % at *p/p*_0_(H_2_O)=0.8 for PCN‐222 and PCN‐223, respectively, of the initial CO_2_ adsorption concentration in pristine PCN‐MOFs. For wetted and pristine PCN‐223, we found an almost identical trend for the competitive CO_2_/H_2_O adsorption (Figure 5d). On the other hand, wetted and pristine PCN‐222 behaved very differently: Firstly, the CO_2_ sorption capacity was increased by 1.8 times after wetting as discussed previously. Secondly, even though the CO_2_ concentration started at higher values, it declined rapidly as soon as water vapor was present. We attributed these differences in CO_2_/H_2_O exchange trends to the difference in pore sizes and the related possibility to form water sorption pockets for CO_2_ (see previous section). In the microporous PCN‐223, no such pockets were formed and we found an almost linear relationship between adsorbed CO_2_ and applied H_2_O vapor concentration for the pristine and wetted PCN‐223. This indicates that H_2_O gradually replaces CO_2_ molecule by molecule. This is in contrast to the mesoporous PCN‐222: The formed water pockets were filled with H_2_O and replaced CO_2_ already at low water vapor pressures. For comparison, we analyzed ZIF‐8, a hydrophobic MOF, with a CO_2_ sorption capacity in the range of PCN‐222 and PCN‐223.[^39^, ^40^] The high hydrophobicity of ZIF‐8 prevented the penetration of water into the pores and only negligible amounts of water were found during the FT‐IR experiment (Figure S24). The ν_as_(C=O) band remained at the same position after wetting compared to the pristine ZIF‐8 (Figure S24) and the CO_2_ sorption capacity was largely unaffected by the presence of water vapor. Finally, the water isotherms of PCN‐222 and PCN‐223 in the presence and absence of CO_2_ (Figure S23) showed a similar trend as in a previous study on the MOF CALF‐20:^[41]^ The water adsorption was influenced by the presence of CO_2_ and shifted the pore condensation onset to higher humidity.

## Conclusions

The uptake and the physicochemical interaction of CO_2_ within the micro‐ and mesopores of porphyrinic Zr‐MOFs were studied in the absence and presence of water vapor. Experimentally collected data from *in situ* FT‐IR and UV‐vis spectroscopy was in line with molecular simulations. Thereby we found the following crucial implications for further studies employing PCN‐MOFs for artificial photosynthesis:

1. *Active site of the photoreaction*. In the absence of water, the CO_2_ sorption sites are close to the Zr‐cluster and stabilized by dispersion forces within the pocket of phenyl‐rings of the linker. However, when water is present in concentrations above the dew point, the sorption site of CO_2_ is shifted away from the Zr‐cluster towards the porphyrin macrocycle. As it is still unclear if the photoenergy is passed to the CO_2_ molecule via an energy transfer from the porphyrin macrocycle or a charge transfer from the Zr‐cluster,[^12^, ^42^] tuning the CO_2_ sorption site intentionally to the vicinity of either site, by simple wetting of the PCN‐MOF, could shine new light on this open question.
2. *Structure and electronic properties of the photosensitizer. In situ* UV‐vis spectroscopy revealed that water interacts with the porphyrin linker and as a consequence a bending of the macrocycle takes place. The bending disrupts the π‐system of the linker and alters the color and thus the spectral overlap of the PCN‐MOFs with the excitation light source used for the artificial photosynthesis. An effect on the energy or charge transfer properties of the photosensitizer can be expected, as it has been observed for uncoordinated porphyrin.[^43^, ^44^] In addition, the basicity of the macrocycle is increased, which can affect the photocatalytic reaction as well.
3. *Competitive adsorption*. CO_2_/H_2_O competitive adsorption experiments at CO_2_ concentrations similar to flue gas (i. e., 15 %) showed that the exchange of CO_2_ with H_2_O is dependent on the MOF pore‐size, hydrophobicity of the MOF material, and on its previous exposure to humidity. The availability of both reaction partners, i. e., CO_2_ and H_2_O, plays an important role in the photoreaction. We showed that the externally applied CO_2_ and H_2_O gas phase concentrations do not directly relate to the CO_2_ and H_2_O concentrations found in the MOF pores and that it depends on the wetting history of the MOFs. However, based on our findings, the CO_2_/H_2_O ratio in the MOF pores can be tuned to desired ratios.
4. *Carbonic acid intermediates*. No experimental evidence for hydrated species of CO_2_, i. e., carbonic acid or carbonates, were found. This indicates that CO_2_ and H_2_O do not react in PCN‐MOFs under the employed experimental conditions.

Overall, these findings can serve as guideline to rationally tune the CO_2_/H_2_O ratio in MOF pores, the photophysical properties of the photosensitizers, as well as the sorption site in the PCN‐MOF pores. This is important to better understand the photoreaction mechanism and identify the catalytic active sites to design efficient and selective photoactive materials for artificial photosynthesis.

## Experimental Section

### Metal‐organic frameworks synthesis and characterization

PCN‐222 was synthesized according to literature.^[18]^ 50 mg 5,10,15,20‐(tetra‐4‐carboxyphenyl)porphyrin (TCPP; 98 %, PorphyChem) and 35 mg ZrCl_4_ (Alfa, 99.5+%) were dissolved in 50 mL dimethylformamide (DMF, TCI, 99 %) using ultrasonic mixing. 14 mL formic acid (FA, TCI, >98 %) were added and the reaction mixture was heated to 120 °C for 16 h in glass vials. After cooling down to room temperature, the purple powder was separated by centrifugation and washed three times with DMF and acetone (AC; VWR, 99.9 %). The powder was dried at 80 °C overnight. PCN‐223 was synthesized according to literature.^[18]^ 50 mg TCPP and 35 mg ZrCl_4_ were dissolved in 50 mL DMF using ultrasonic mixing. 10 mL propionic acid (PA; Merck,99 %) were added and the reaction mixture was heated to 120 °C for 16 h in glass vials. After cooling down to room temperature, the purple powder was separated by centrifugation and washed three times with DMF and acetone. Subsequently, the MOF powders were soaked in acetone for a day and separated by centrifugation. The powder was dried at 80 °C overnight. 1 mg of MOF powder was responded in 0.2 mL acetone and the suspension was drop casted on the Si ATR crystal heated at 80 °C. ZIF‐8 (500 nm crystal size) was purchased at ACS materials.

UV‐vis spectra of MOF suspensions in water, acetone, toluene (VWR, 99.9 %) and 0.01 M HCl (Honeywell, p.a.) were determined using a Lambda 950S UV‐vis‐NIR spectrophotometer (PerkinElmer) equipped with a deuterium and a halogen light source, a photomultiplier (PMT) detector (UV‐vis region), and a 150 mm integrating sphere coated with Spectralon®. Suspensions were filled in quartz cuvettes with d=2 mm and placed in front of the integrating spere. X‐ray diffraction (XRD) data were collected on a Bruker D8 Advance X‐ray powder diffractometer in Bragg‐Brentano geometry operating with a Cu K_α_ X‐ray tube (λ=1.5418 Å) at 40 kV and 40 mA. Powder samples were ground and placed on a silicon single crystal sample holder. XRD patterns were recorded at room temperature between 2° to 25° at a rate of 1 s/step and a step size of 0.02°. N_2_ isotherms were recorded on a Sync 400 (3P Instruments) using N_2_ (Linde, 5.0). Samples were activated under a dynamic vacuum at 150 °C for 12 h prior the measurement. A liquid N_2_ bath was used to maintain a temperature of 77 K for each measurement.

### Experimental setup for gas sorption

Figure S1 shows the gas flow cell and attenuated total reflection (ATR) setup that was placed in a Perkin Elmer Three Fourier‐transform infrared (FT‐IR) spectrometer equipped with a N_2_‐cooled mercury cadmium telluride (MCT) detector, which has been adapted from Ref. [45,46]. 1/8 inch fluoropolymer (PFA) tubing was connected to the gas flow cell using M5 adapters (Festo). For each spectrum 128 scans were averaged (1 min/spectrum). Attenuated total reflectance (ATR) crystals (20×10×0.5 mm, 45°) cut from double side polished Si wafer and a depth of penetration *d_p_* =0.52 μm (ν=1600 cm^−1^) and an effective pathlength of $d_{e∥}$ =0.64 μm $d_{e⊥}$ =0.32 μm, yielding at total effective pathlength of ($d_{e∥}$ +$d_{e⊥}$ )/2*N=9.65 μm with N=20 were used. The temperature of the aluminium flow cell was controlled using a thermoelectric cooling (TEC) controller (TEC‐1091, Meerstetter) and a 20×20 mm Peltier element.

CO_2_ mixtures were obtained by mixing pure CO_2_ (Linde, N.4) and N_2_ (Linde, 5.0) streams. Water vapor with different *p/p*_0_(H_2_O) was generated by mixing a dry N_2_ flow with a moistened N_2_ flow obtained from bubbling through water at room temperature by means of mass flow controllers (Bronkhorst) to achieve a total flow of 100 mL min^−1^. The actual water vapor concentrations were determined by transmission IR spectroscopy in a 10 cm transmission cell with ZnSe windows. For calibration, transmission spectra were integrated in the O−H bending region between 1700–1800 cm^−1^. Concentrations were obtained from the band areas using reference spectra of 1 ppm/m water from the PNNL database. The application sequence was started with flushing pure N_2_ flushing for 10 min. Subsequently, the partial pressure was increased/decreased and kept for 3 min to reach step to reach equilibrium.

Fiber‐coupled UV‐vis spectroscopy in diffuse reflectance (DR) mode from the Si ATR crystal was performed using an Avantes Avaspec‐ULS2048CL‐EVO‐RS spectrometer and an AvaLight‐DH‐S‐BAL light source. A reflection probe from Avantes (FCR‐7UV400) was placed above the flow cell to measure through the sapphire window.

### Computational details

The calculations were done with the ADF program, versions 2020.102 and 2022.101^[47][48]^ using a double‐ζ plus polarization function [DZVP] basis set.^[49]^ Relativistic effects were accounted for by the Zeroth Order Regular Approximation (ZORA) approach.[^50^, ^51^, ^52^] We have used the meta‐GGA M06‐L density functional;^[53]^ all electrons were included in the calculations (i. e., no frozen cores or effective core potentials were used). The numerical accuracy setting of the ADF program was specified as “good”. The standard ADF criteria for geometry optimization were applied. For the clusters representing the MOFs, the carbon atoms in *para* position to the carboxylate groups were fixed at the positions in the solid. The reported sorption energies were corrected for the Basis Set Superposition Error (BSSE) by means of the counterpoise method.^[54]^ For calculating vibrational frequencies we used the Mobile Block Hessian method.[^55^, ^56^]

## Supporting Information

Characterization (N_2_ sorption, FT‐IR spectroscopy, and X‐ray diffraction) of MOFs; computational details, supplemental FT‐IR and UV‐vis DRS data of pristine and wetted PCN‐MOFs are provided in the Supporting Information. The authors have cited additional references within the Supporting Information.[^57^, ^58^, ^59^, ^60^, ^61^, ^62^]

## Conflict of interest

The authors declare no conflict of interest.

1

## Supporting information

As a service to our authors and readers, this journal provides supporting information supplied by the authors. Such materials are peer reviewed and may be re‐organized for online delivery, but are not copy‐edited or typeset. Technical support issues arising from supporting information (other than missing files) should be addressed to the authors.

Supporting Information

## Data Availability

The data that support the findings of this study are available from the corresponding author upon reasonable request.
