# Supplementary material for: The Role of Water in Carbon Dioxide Adsorption in Porphyrinic Metal‐Organic Frameworks
Source: ChemCatChem. 2023 Aug 8;15(19):e202300722. doi: 10.1002/cctc.202300722 (PMC10946852; doi:10.1002/cctc.202300722)
Supplement: Supplementary file 1 — Supporting Information [file CCTC-15-0-s001.pdf]

ChemCatChem

Supporting Information

The Role of Water in Carbon Dioxide Adsorption in Porphyrinic Metal-Organic Frameworks

Bettina Baumgartner, P. Tim Prins, Jaap N. Louwen, Matteo Monai, and Bert M. Weckhuysen*

Content

Experimental procedures 2

Experimental setup for gas sorption 2

Results and discussion 3

Characterization of metal-organic frameworks 3

Calculation of CO_2 sorption capacity from IR absorbance 5

In situ spectroscopy of CO_2 adsorption into pristine metal-organic frameworks 6

In situ spectroscopy of CO_2 adsorption into wetted metal-organic frameworks 12

In situ visible spectroscopy of wetted metal-organic frameworks 15

References 20

Author contributions 21

Experimental procedures

Experimental setup for gas sorption

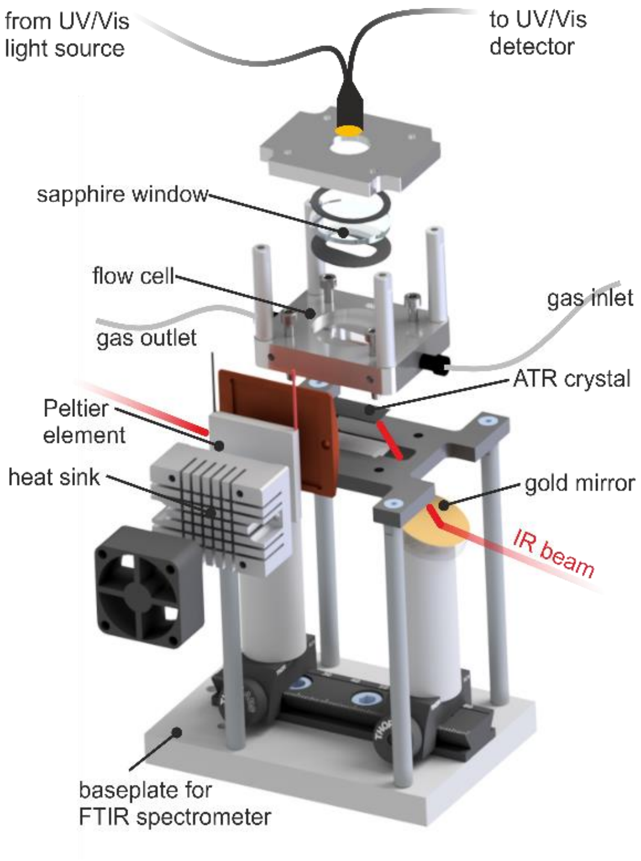

Figure S1. 3D rendering of the experimental setup.

Results and discussion

Characterization of metal-organic frameworks

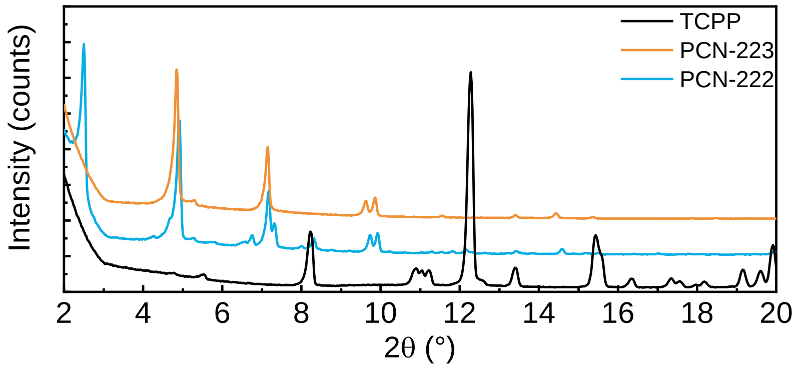

Figure S2. X-ray diffraction (XRD) pattern of TCPP, PCN-222, and PCN-223.

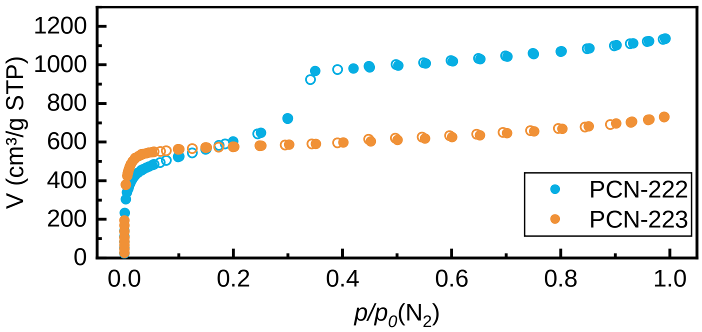

Figure S3. N_2 sorption of PCN-222 and PCN-223 yielding Brunauer-Emmett-Teller (BET) surface areas of 2100 m^2 g^{-1} and 2340 m^2 g^{-1} for PCN-222, and PCN-223, respectively.

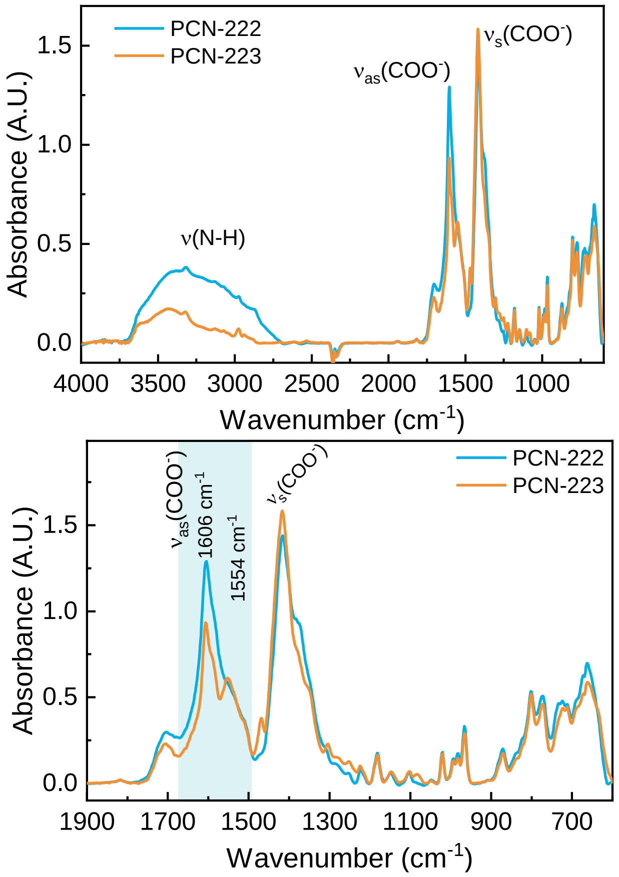

Figure S4. Fourier transform-infrared (FT-IR) spectra of 2 wt% PCN-222 and PCN-223 in KBr pellets recorded in transmission. Left: full range FT-IR spectra, Right: zoom into fingerprint region.

Calculation of CO_2 sorption capacity from IR absorbance

Determine mass fraction of MOFs and linker present on in the evanescent wave on the ATR crystal via KBr reference measurement using the effective pathlength de. Thereby, following volume fractions were determined:

PCN-222	2 %
PCN-223	5 %
TCPP	13 %
ZIF-8	85 %

With the given geometrical volume of the evanescent wave (d_e x 1 cm x 2 cm) and the volume fractions, the volume of the samples on the ATR crystal in cm^3 was determined.

The concentration of CO_2 in mmol L^{-1} present in the evanescent field was derived using de and assuming that absorption coefficient for dissolved CO_2 in water (54.2 L g^{-1} cm^{-1}) is similar as condensed CO_2 in MOF

The CO_2 sorption capacity in $mmol_{CO2}/g_{MOF}$ was calculated using the crystal density of the MOF of 0.52 g cm^{-3}.

In situ spectroscopy of CO_2 adsorption into pristine metal-organic frameworks

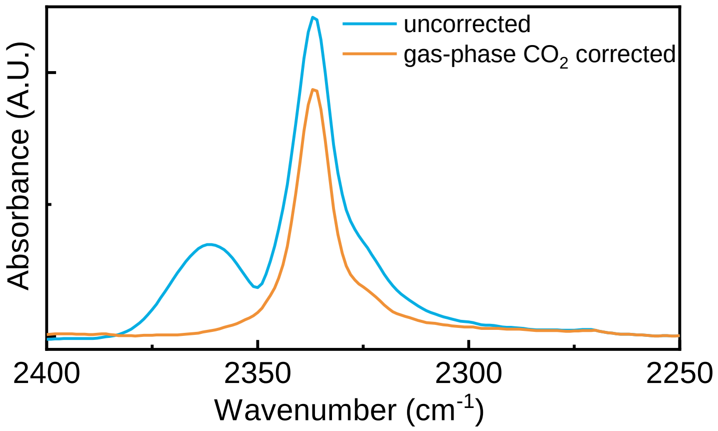

Figure S5. The CO_2 band area was corrected by subtracting the gas phase CO_2 spectrum recorded using the same setup (i.e., the attenuated total reflectance (ATR) flow cell).

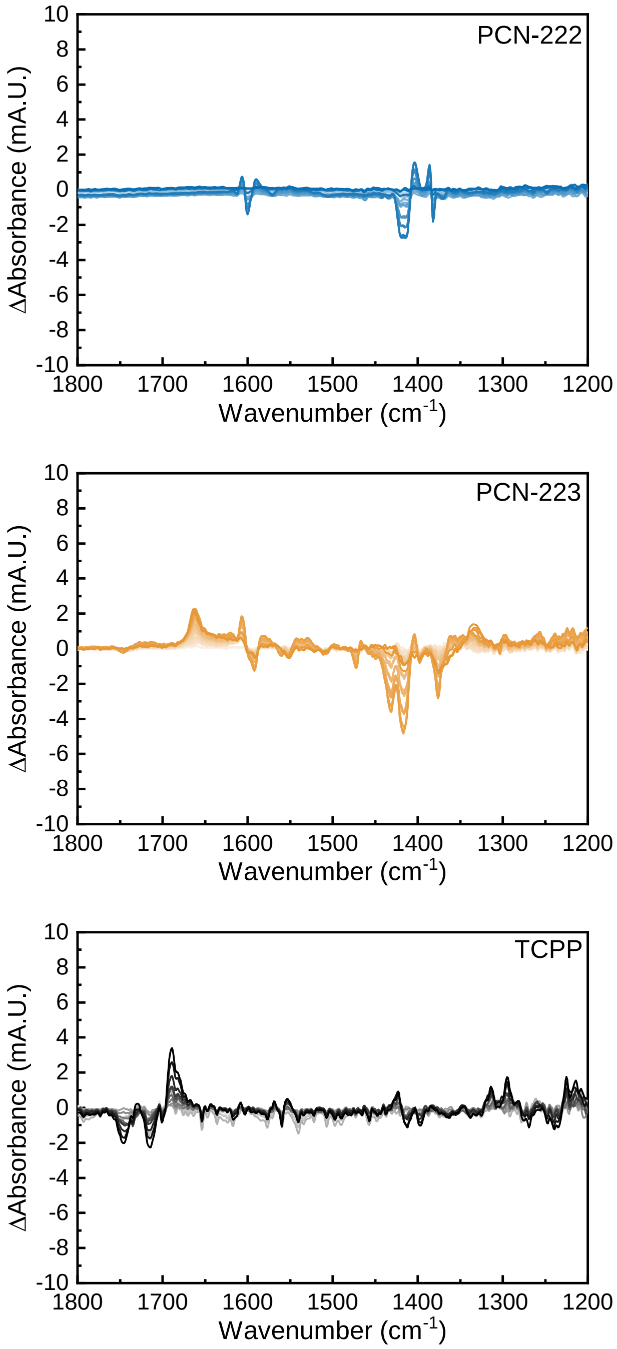

Figure S6. Fingerprint region of difference Fourier transform infrared (FT-IR) spectra of PCN-222, PCN-223, and TCPP upon CO_2 sorption between $p(CO_2)$ = 0.02 - 0.75 bar. Spectra of corresponding samples show band heights > 100 mA.U. and therefore, the shown spectral changes correspond to >1 % of changes in the samples.

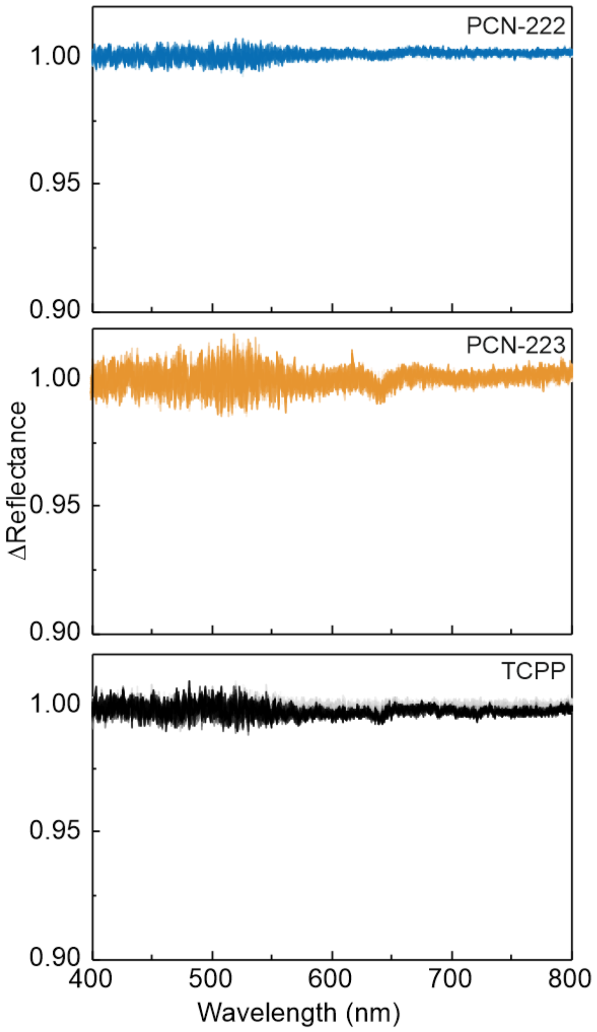

Figure S7. Difference UV-vis reflectance spectra of PCN-222, PCN-223, and TCPP upon CO_2 sorption with $p(CO_2)$ = 0.75 bar. Spectra were recorded every second during the exchange from N_2 to CO_2 into pristine metal-organic frameworks (MOFs) and linker.

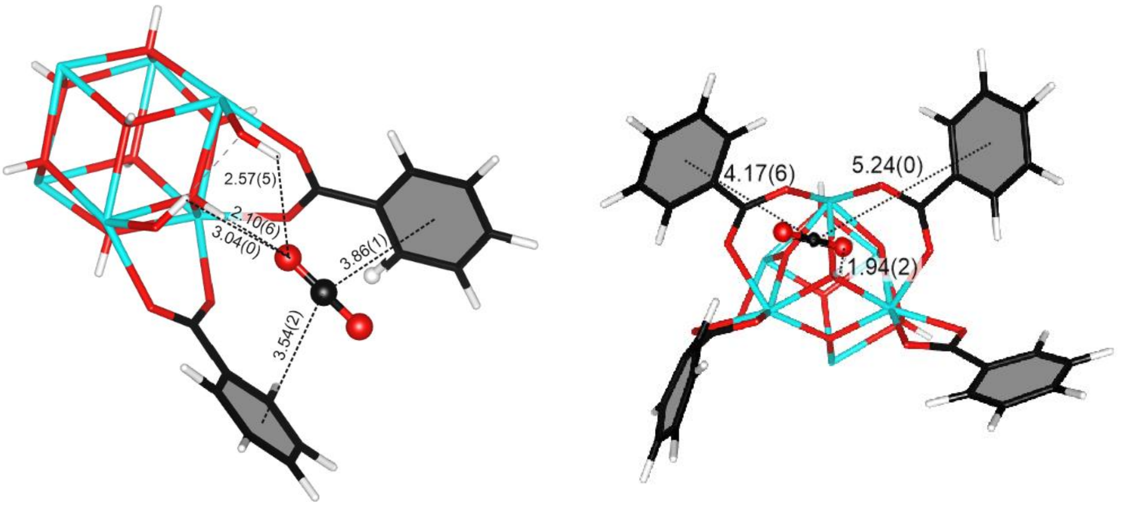

Figure S8. Views of binding of CO_2 in PCN-222 and PCN-223. All units are quoted in Å. (Zr, aqua; C, black, O, red; H, white).

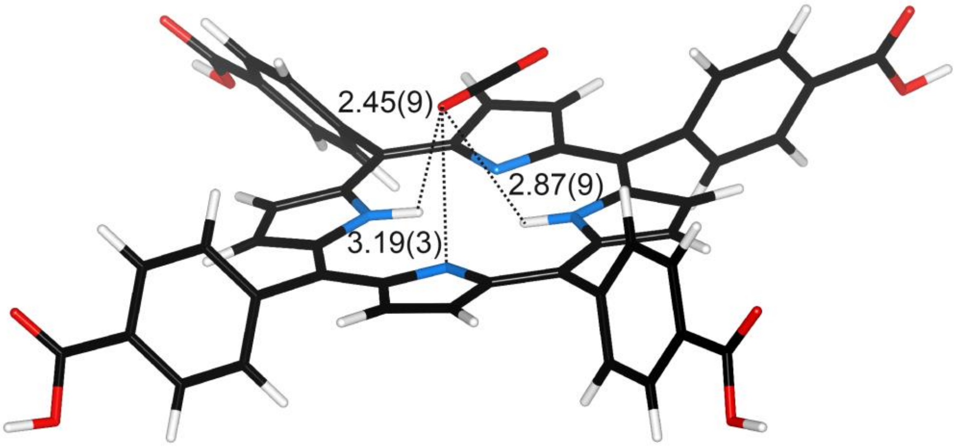

Figure S9. Views of binding of CO_2 on porphyrin linker. All units are quoted in Å. (N, blue; C, black, O, red; H, white).

To gauge the importance of the respective contributions of hydrogen bonding and Van der Waals attraction, we took the complex of the PCN-222 cluster with CO_2 and replaced the two phenyl rings around CO_2 by hydrogen atoms. The positions of these two hydrogen atoms were optimized, with the positions of all other atoms kept fixed. We find that the energy of interaction between the CO_2 and cluster fragments in this new complex is - 17 kJ mol^{-1}, which indicates that about half the adsorption energy computed for CO_2 on PCN-222 is due to dispersion interaction with the phenyl rings, the other half being due to interaction with the cluster – mainly hydrogen bonding.

The influence of a second CO_2 molecule on the sorption enthalpy and sorption site of the first CO_2 molecules was simulated. We found that the adsorption of a second CO_2 molecule does not take place in the same phenyl-ring envelop as the first CO_2 molecule and therefore does not influence the sorption site or sorption enthalpy of the first CO_2 molecule that we investigate in this study (Figure S10).

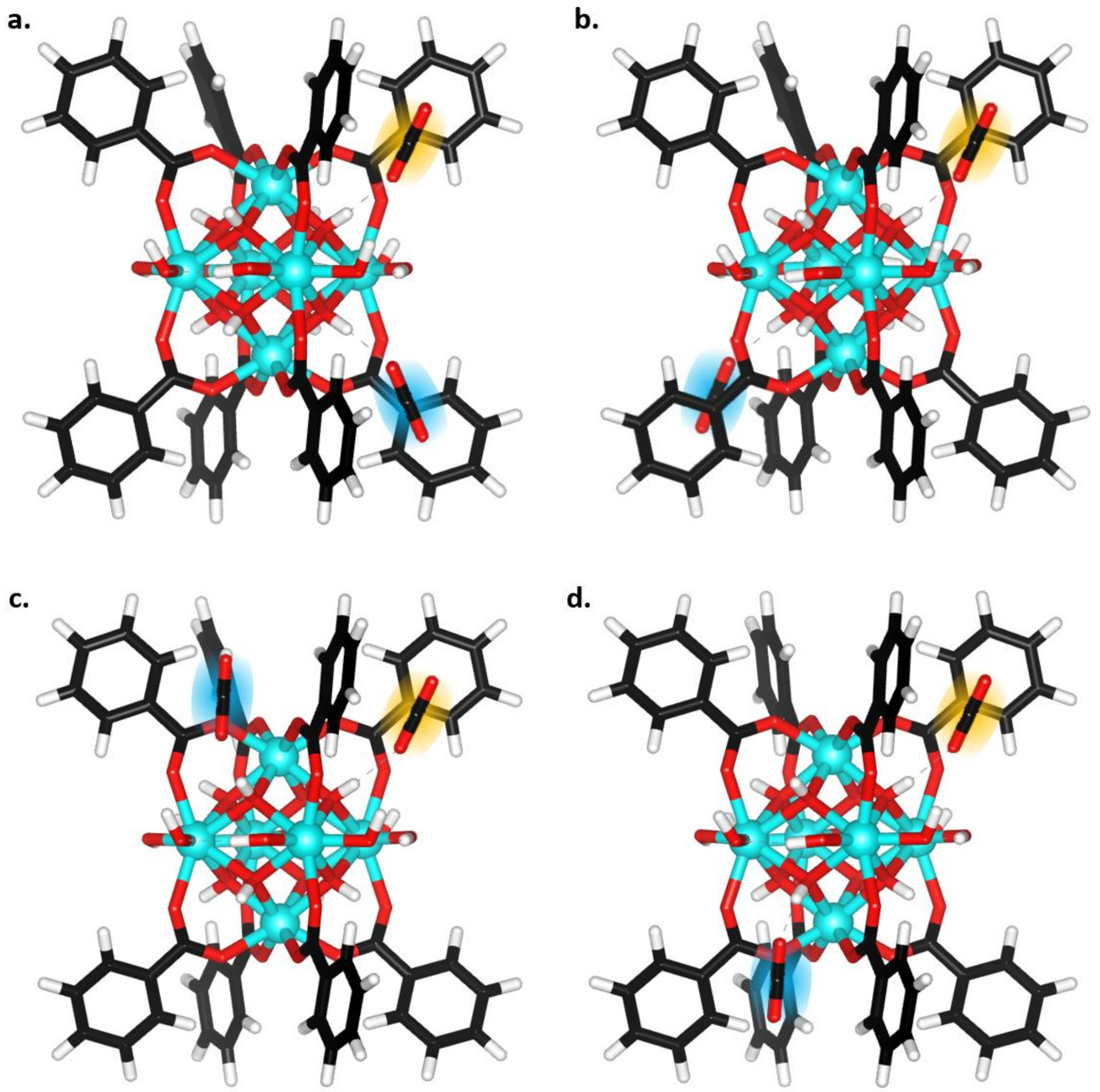

Figure S10. Views of binding of a second CO_2 molecule (highlighted in blue) adsorbed to the Zr-cluster of PCN-222 in addition the first CO_2 molecule (highlighted in yellow) as discussed in the main text. (N, blue; C, black, O, red; H, white). Density functional theory simulations yielded that the adsorption enthalpy, and thus the sorption site, of the first CO_2 molecule is not affected by the second CO_2 molecule for all investigated scenarios (a.-d.). However, we found that sorption between the phenyl rings of the TCPP linker that are closer to each other (scenario a. and b.) was more favorable then sorption between two rings further apart (c. and d.).

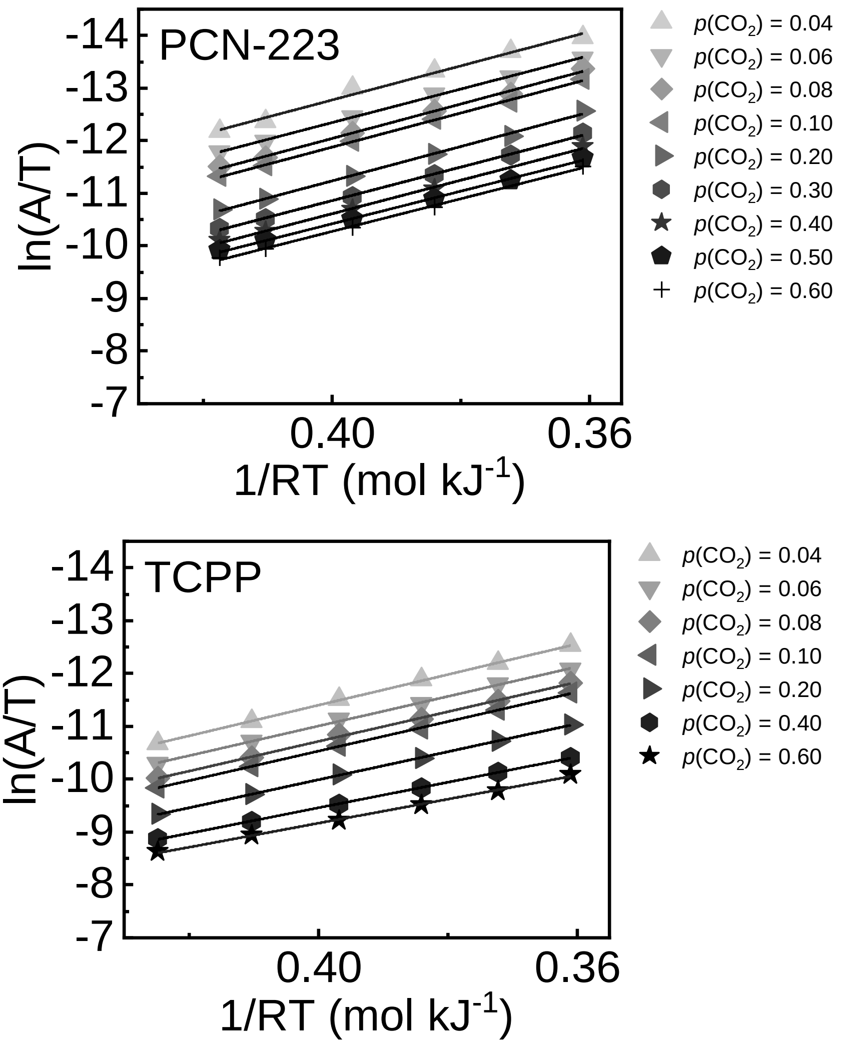

Figure S11. Linearized plot of CO_2 adsorbed into PCN-223 and TCPP for $p(CO_2)$ = 0.04 bar -0.75 bar at 15-60 °C used for deriving the adsorption enthalpy using the van 't Hoff equation.

In situ spectroscopy of CO_2 adsorption into wetted metal-organic frameworks

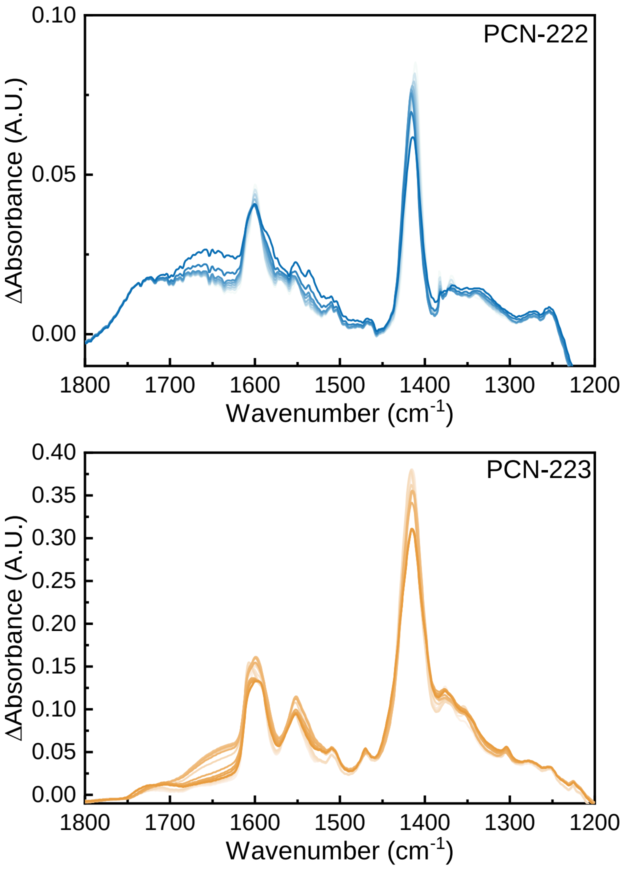

Figure S12. Fourier transform-infrared (FT-IR) spectra of PCN-222 and PCN-223 upon water sorption between $p/p_0(H_2O)$ = 0 – 0.8. (bright to dark traces). Background = blank Si attenuated total reflectance (ATR) crystal. Note that in contrast to gas-phase CO_2, no sharp water vapour IR bands were observed as absolute water vapour concentrations were lower and the absorption coefficient of water is lower compared to CO_2.

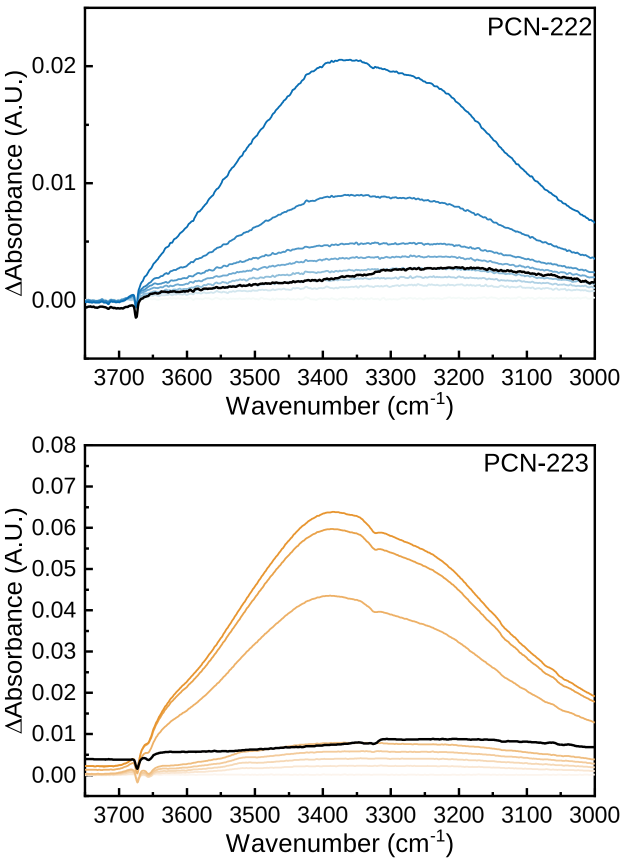

Figure S13. Fourier transform-infrared (FT-IR) spectra of PCN-222 and PCN-223 upon water sorption zoom to Zr-OH region. black spectra = after N_2 flushing for 10 min. Background = first scan. An additional Zr-OH band at 3655 cm^{-1} is present in the FT-IR spectrum of PCN-223.[1]

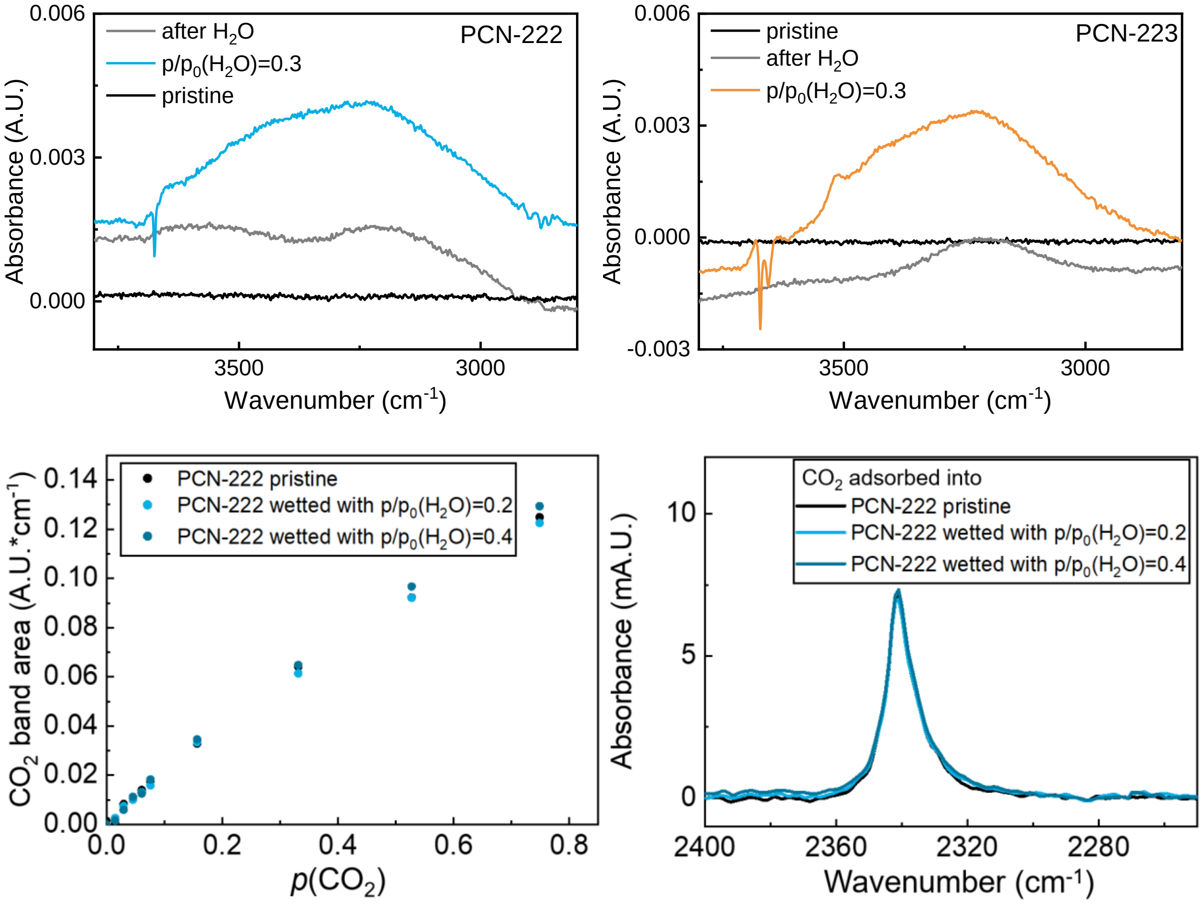

Figure S14. Partial wetting experiments: Top: Fourier transform-infrared (FT-IR) difference spectra of dry, $p/p_0(H_2O)$=0.3, and dried metal-organic frameworks (MOFs). Bottom: sorption capacity of PCN-222 after wetting and corresponding ν(C=O) bands.

In situ visible spectroscopy of wetted metal-organic frameworks

We suspected two phenomena to be responsible for the obtained UV-vis difference reflectance spectra: i) A shift of the electronic bands due to the change in the sample refractive index during pore water condensation, and/or ii) the protonation of all pyrrole moieties in the porphyrin, and an associated bending of the macrocycle that has been observed for the TCPP linker and PCN-222 upon acidification.[2–4] To test for hypothesis (i), we have collected UV-vis reference spectra of the MOFs in suspensions of aprotic solvents having different refractive indexes. We selected acetone and toluene ($n_{acetone}$ = 1.35, $n_{toluene}$=1.49)[5,6], to simulate the change in refractive index from air to water in the MOF pores (n_{air} = 1.00, n_{water}=1.33), while avoiding protonation.[7,8] To test for hypothesis (ii), we collected UV-vis reference spectra of MOFs in a 0.01 M HCl water suspension, which is known to protonate the porphyrin, and to affect the π-system of the macrocycle, inducing a color shift from purple to green (Figure S14). The thereby observed differential UV-vis absorptance spectra are given in Figure S16. For both MOFs, the change of refractive index replicated the majority of the features but did not account for the negative band at 650 – 700 nm, which originated from the protonated form of the TCPP.[2–4,9] This feature was only visible in the spectrum of MOFs in the 0.01 M HCl solution. These findings suggest that both events, i.e., band shifts due to the increase in refractive index and porphyrin bending take place upon pore condensation. To account for both events, a difference spectrum was calculated from the measurements using 0.01 M HCl and toluene and this resembled the obtained *in situ* data best (see Figure 4c, dashed line).

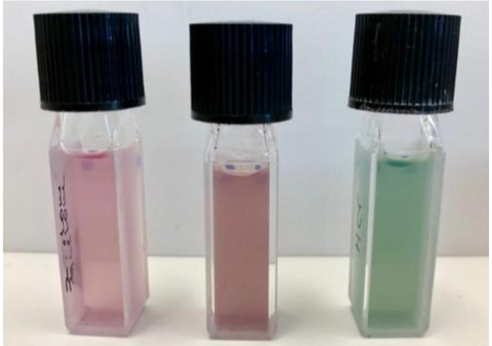

Figure S15. Photographs of PCN-223 suspended in acetone, water and 0.01 M HCl from left to right.

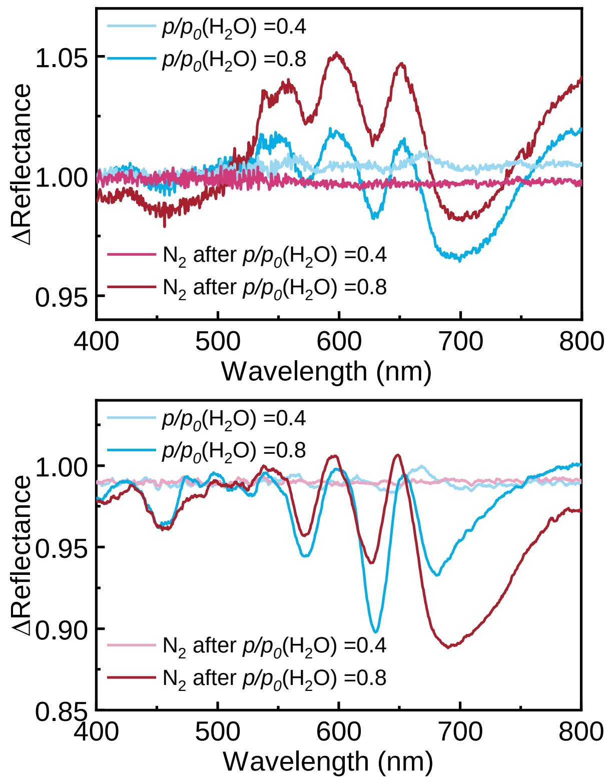

Figure S16. Difference *in situ* UV-vis diffuse reflectance spectra of PCN-222 (left) and PCN-223 (right) for different $p/p_0(H_2O)$.

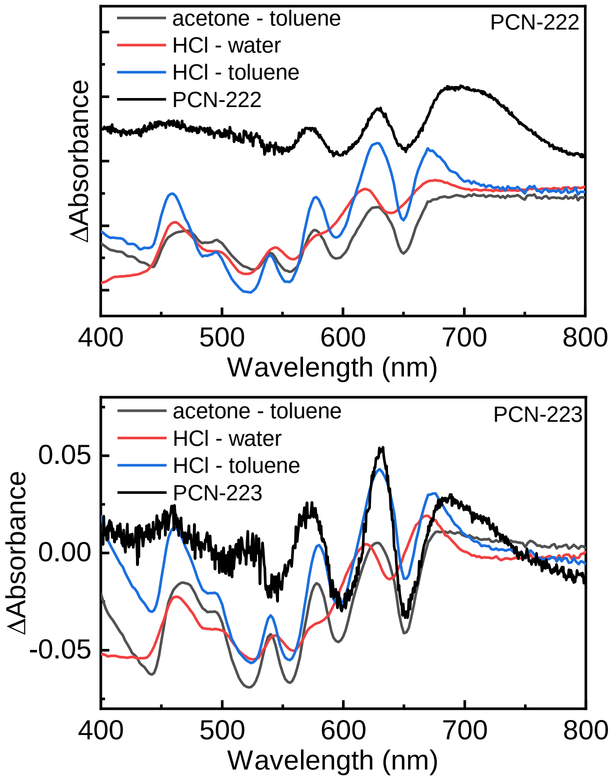

Figure S17. Difference UV-vis absorptance spectra of PCN-222 and PCN-223 overlaid with reference UV-vis spectra obtained from metal-organic framework (MOF) suspensions in water, acetone, toluene or 0.01 M HCl.

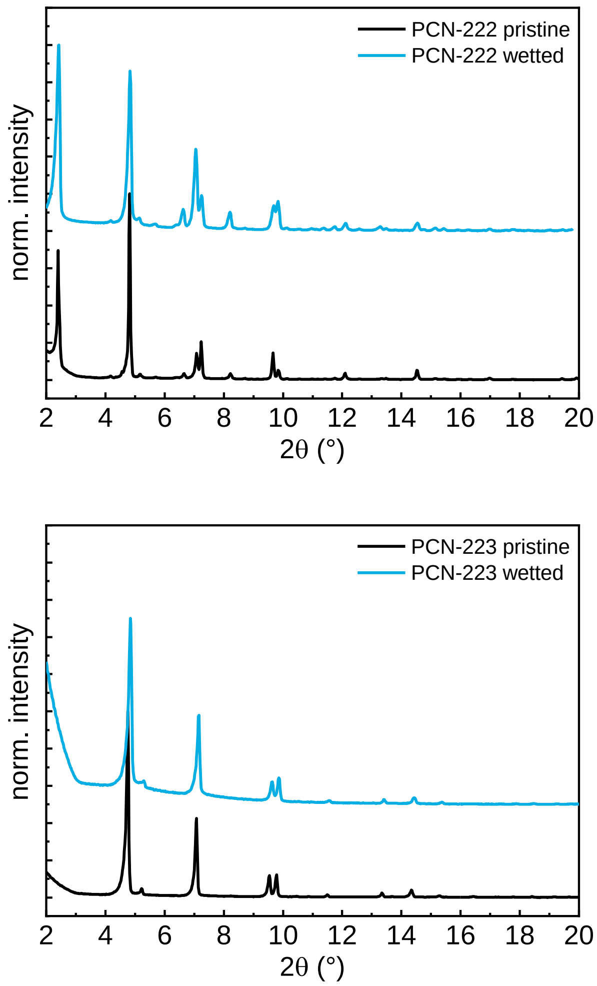

Figure S18. X-ray diffraction (XRD) patterns of dry and wetted PCN-222 and PCN-223.

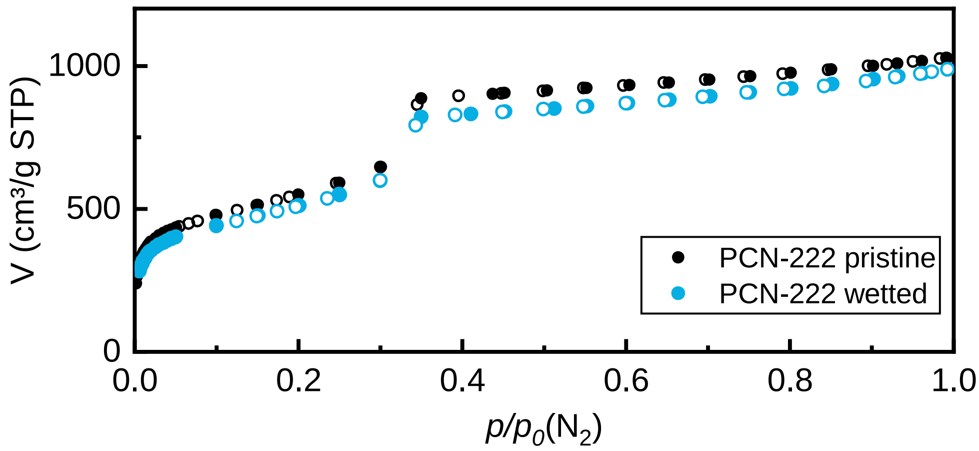

Figure S19. N_2 physisorption of dry and wetted PCN-222.

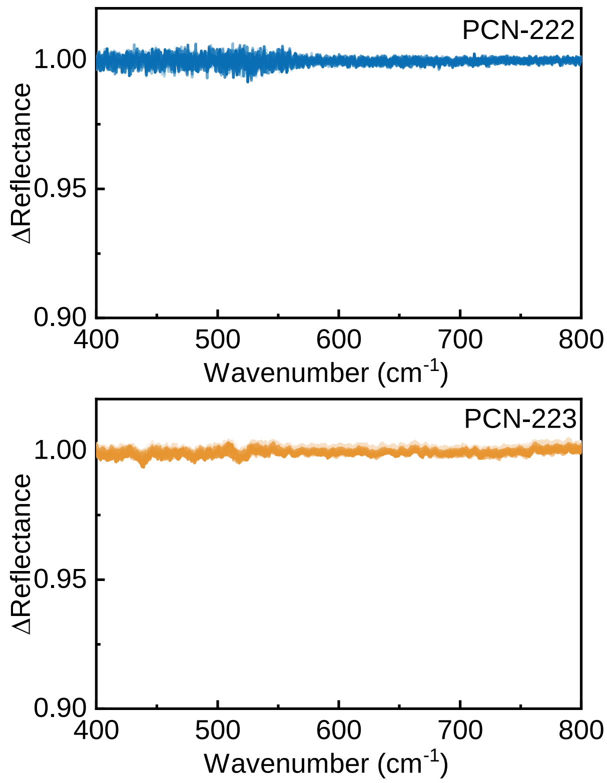

Figure S20. Difference UV-vis diffuse reflectance spectra of PCN-222 and PCN-223 during CO_2 adsorption ($p(CO_2)$=0.75 bar) after wetting.

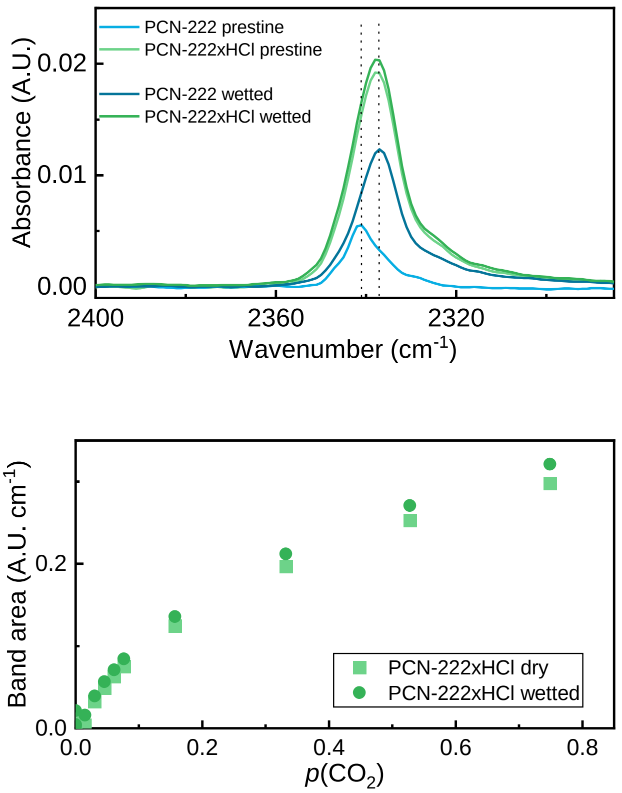

Figure S21. CO_2 sorption into acidified PCN-222. To this end, the PCN-222 coated attenuated total reflection (ATR) crystal was flushed with liquid 0.01 M HCl prior the placement in the flow cell.

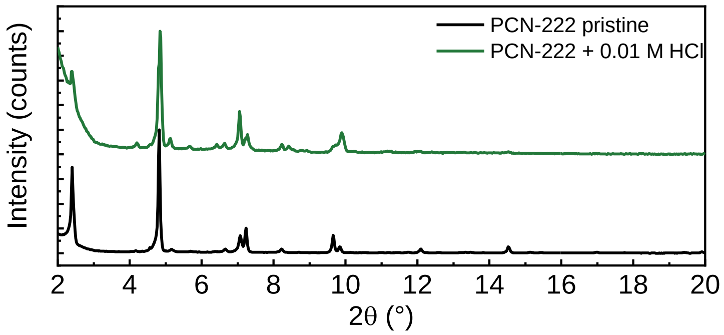

Figure S22. X-ray diffraction pattern of pristine and PCN-222 acidified with 0.01 M HCl.

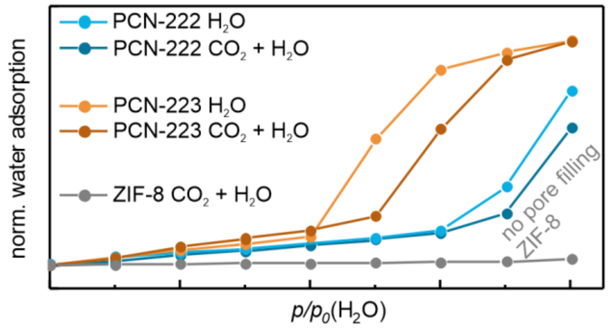

Figure S23. Water adsorption into pristine MOFs and in the presence of $p(CO_2)$ = 0.15 bar. Water adsorption was influenced by the presence of CO_2 and showed a shift of the pore condensation onset for PCN-223 from $p/p_0(H_2O)$ = 0.4 to 0.5, while the effect was less pronounced for PCN-222.

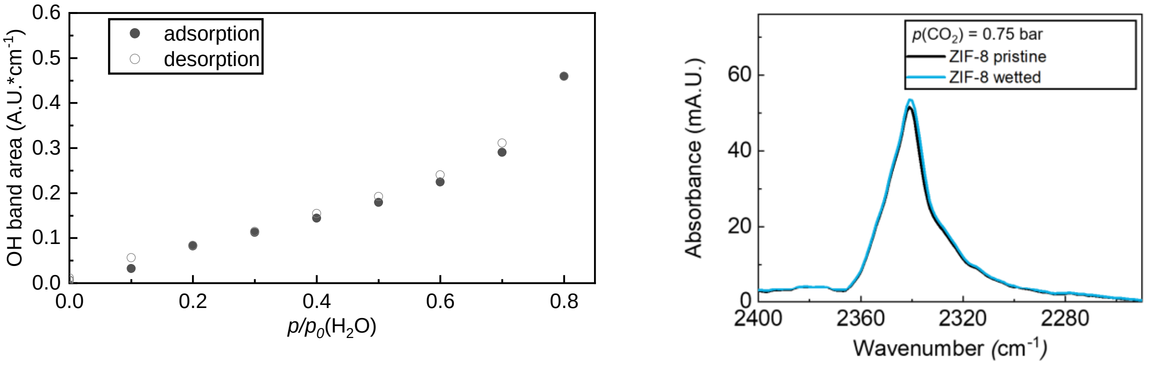

Figure S24. (left) water isotherm of ZIF-8. Type 3 isotherm is typical for hydrophobic materials. (right) CO_2 band obtained from pristine and wetted ZIF-8.

References

1. Cavka, J. H., Jakobsen, S., Olsbye, U., Guillou, N., Lamberti, C., Bordiga, S. & Lillerud, K. P. A new zirconium inorganic building brick forming metal organic frameworks with exceptional stability. *J. Am. Chem. Soc.* **130**, 13850–13851 (2008).

2. Smith, K. T., Ramsperger, C. A., Hunter, K. E., Zuehlsdorff, T. J. & Stylianou, K. C. Colorimetric detection of acidic pesticides in water. *Chem. Commun.* **58**, 953–956 (2022).

3. Deibert, B. J. & Li, J. A distinct reversible colorimetric and fluorescent low pH response on a water-stable zirconium–porphyrin metal–organic framework. *Chem. Commun.* **50**, 9636–9639 (2014).

4. Shaikh, S. M., Chakraborty, A., Alatis, J., Cai, M., Danilov, E. & Morris, A. J. Light harvesting and energy transfer in a porphyrin-based metal organic framework. *Faraday Discuss.* **216**, 174–190 (2019).

5. Kedenburg, S., Vieweg, M., Gissibl, T. & Giessen, H. Linear refractive index and absorption measurements of nonlinear optical liquids in the visible and near-infrared spectral region. *Opt. Mater. Express* **2**, 1588 (2012).

6. Rheims, J., Köser, J. & Wriedt, T. Refractive-index measurements in the near-IR using an Abbe refractometer. *Meas. Sci. Technol.* **8**, 601–605 (1997).

7. Hale, G. M. & Querry, M. R. Optical Constants of Water in the 200-nm to 200-µm Wavelength Region. *Appl. Opt.* **12**, 555 (1973).

8. Peck, E. R. & Khanna, B. N. Dispersion of Nitrogen. *J. Opt. Soc. Am.* **56**, 1059 (1966).

9. Presselt, M., Dehaen, W., Maes, W., Klamt, A., Martínez, T., Beenken, W. J. D. & Kruk, M. Quantum chemical insights into the dependence of porphyrin basicity on the meso-aryl substituents: Thermodynamics, buckling, reaction sites and molecular flexibility. *Phys. Chem. Chem. Phys.* **17**, 14096–14106 (2015).

Author contributions

BB: data curation, formal analysis, investigation, writing of original draft, funding acquisition

PTP: data curation, investigation, supporting writing of original draft

JNL: formal analysis, investigation, supporting writing of original draft

MM: data curation, supporting writing of original draft

BMW: data curation, funding acquisition, supporting writing of original draft, supervision
